# Sequential receptor engagement dictates the broad host range and fitness trade-offs of *Salmonella* phage PSA5-1

**DOI:** 10.1128/spectrum.00248-26

**Published:** 2026-05-29

**Authors:** Muhammad Saleem Iqbal Khan, Yuan Li, Shenlin Ji, Shuangshuang Hou, He Li, Yaoyuan Chang, Demeng Tan, Ju Wu, Jiajun Yin

**Affiliations:** 1 Department of General Surgery, Affiliated Zhongshan Hospital of Dalian University, Dalian, Liaoning, China; 2 Shanghai Public Health Clinical Center, Fudan University 12478, Shanghai, China; CEB - Centre of Biological Engineering, Universidade do Minho, Braga, Portugal

**Keywords:** *Salmonella enterica*, bacteriophage, phage–host interaction, phage receptors

## Abstract

The global rise of multidrug-resistant (MDR) *Salmonella* spp. necessitates the development of alternative antimicrobials, yet the molecular determinants of phage host range and the collateral effects of bacterial resistance remain poorly defined. Here, we characterize PSA5-1, a T4-like phage, and decipher the hierarchical two-step receptor recognition governing its infection of *Salmonella enterica*. We demonstrate that PSA5-1 utilizes the outer-membrane porin C (OmpC) as a primary receptor required for reversible adsorption, while the lipopolysaccharide (LPS) inner core is required for productive infection. Selection for PSA5-1 resistance reveals distinct evolutionary trajectories: whereas *ompC* deletion confers resistance with minimal fitness costs, disruption of the LPS core (via *rfaF*) triggers profound physiological remodeling and enhanced biofilm formation. Comparative genomics further indicates that host susceptibility is governed by OmpC sequence conservation and O-antigen diversity, with additional contributions from intracellular defense systems. By mapping the interplay between receptor engagement and resistance-associated trade-offs, our findings provide a mechanistic framework for the rational design of precision phage-based interventions against enteric pathogens.

**IMPORTANCE:**

The increasing prevalence of multidrug-resistant *Salmonella* spp. necessitates the development of precision antimicrobial alternatives to antibiotics, such as bacteriophage therapy. However, the successful clinical application of phages is often hampered by the rapid evolution of bacterial resistance and complex host defense mechanisms. In this study, we dissect the infection logic of the phage PSA5-1, identifying the outer-membrane protein C–lipopolysaccharide inner core dependency as a critical determinant enabling sequential receptor engagement during genome delivery. By mapping the genetic determinants of susceptibility, ranging from receptor variation to intracellular immunity, we highlight the evolutionary trade-offs *Salmonella* must navigate to evade infection. These findings provide a mechanistic blueprint for selecting phages that exploit conserved surface targets, ultimately aiding in the design of therapeutic cocktails that are robust against bacterial resistance.

## INTRODUCTION

The interaction between bacteriophages and their bacterial hosts is a dynamic and intricate process that plays a pivotal role in shaping microbial ecology, evolution, and population fitness (1). Among bacteriophages, T4-like members of the order *Caudovirales* are structurally complex and among the most extensively studied bacteriophages (2, 3). Phage infection begins with adsorption mediated by receptor-binding proteins (RBPs), which are incorporated into the distal tail fibers or spikes to recognize specific receptor molecules on the bacterial cell surface, initiating host recognition and genome delivery (4). This initial recognition event is both a determinant of host range and a key factor influencing the therapeutic potential of phages as precision antibacterial agents (5, 6). Despite this, our understanding of the molecular and genetic determinants that govern host–phage interactions remains incomplete, even in well-studied model systems (6–9).

In Gram-negative bacteria, particularly in *Salmonella enterica*, the cell surface presents a highly diverse repertoire of potential phage receptors. These include outer membrane proteins (OMPs) and lipopolysaccharide (LPS) components, including O-antigen repeats and the core oligosaccharide (inner and outer core), along with various proteinaceous appendages, such as pili, fimbriae, and flagella (10–12). Each of these structures plays a dual role: they are integral to bacterial physiology, fitness, and virulence, yet simultaneously act as molecular gateways for phage infection. While classical *Salmonella* phages, such as P22, Epsilon15, Det7, Felix O1, and S16, typically utilize LPS or OmpC as principal receptors (10, 13–15), other phages are known to engage alternative surface structures. These include type one fimbriae or capsular polysaccharides (such as the Vi antigen in *Salmonella* Typhi) for initial adsorption and subsequent genome delivery (16, 17).

This structural and functional diversity of surface receptors underpins the evolutionary arms race between phages and their hosts (18, 19). While many *Salmonella* phages display strict monoreceptor specificity, recent studies have identified polyvalent phages within the T4 superfamily (13). Compared with classic T-even phages (T2, T4, and T6), some polyvalent phages possess broad host ranges by engaging multiple host surface components. They achieve initial, reversible binding to the host using hypervariable long tail fibers (LTFs) or receptor-binding proteins (RBPs) via a gp38 adhesion protein located at their tips. Subsequently, short tail fibers (STFs) facilitate secondary, irreversible binding to maximize infection efficiency and sustain their broad host range (20–23). Notably, a single RBP can recognize multiple distinct receptors, as demonstrated for T4 gp37, which binds both LPS and OmpC through separate surface regions (24). Such versatility confers a selective advantage by reducing the likelihood of resistance emergence and mitigating the fitness costs typically associated with receptor loss or modification (25). Building on this concept, phage cocktails combining complementary receptor specificities, such as those targeting LPS, flagella, and OMPs, have demonstrated improved efficacy and durability against *Salmonella* infections. Such receptor-informed cocktail designs represent a promising avenue for the development of broad-spectrum and evolution-resilient phage therapies (10).

Beyond receptor-level specificity and blocking, the broader bacterial genomic landscape plays a decisive role in determining phage susceptibility (26). Comparative genomic and genome-wide screening studies have revealed that bacterial genomes harbor dynamic defense hotspots, where mobile genetic elements (MGEs), prophages, and antiphage systems, such as restriction–modification (RM), BREX, and retron modules cluster to form adaptive “defense islands.” These elements collectively shape the bacterial evolutionary response to phage predation (8, 27, 28). Integrating receptor biology with genomic defense profiling is thus essential to unravel how surface-level recognition events interact with intracellular immunity to define host range, infection outcomes, and resistance trajectories in *Salmonella*.

**Fig 1:**
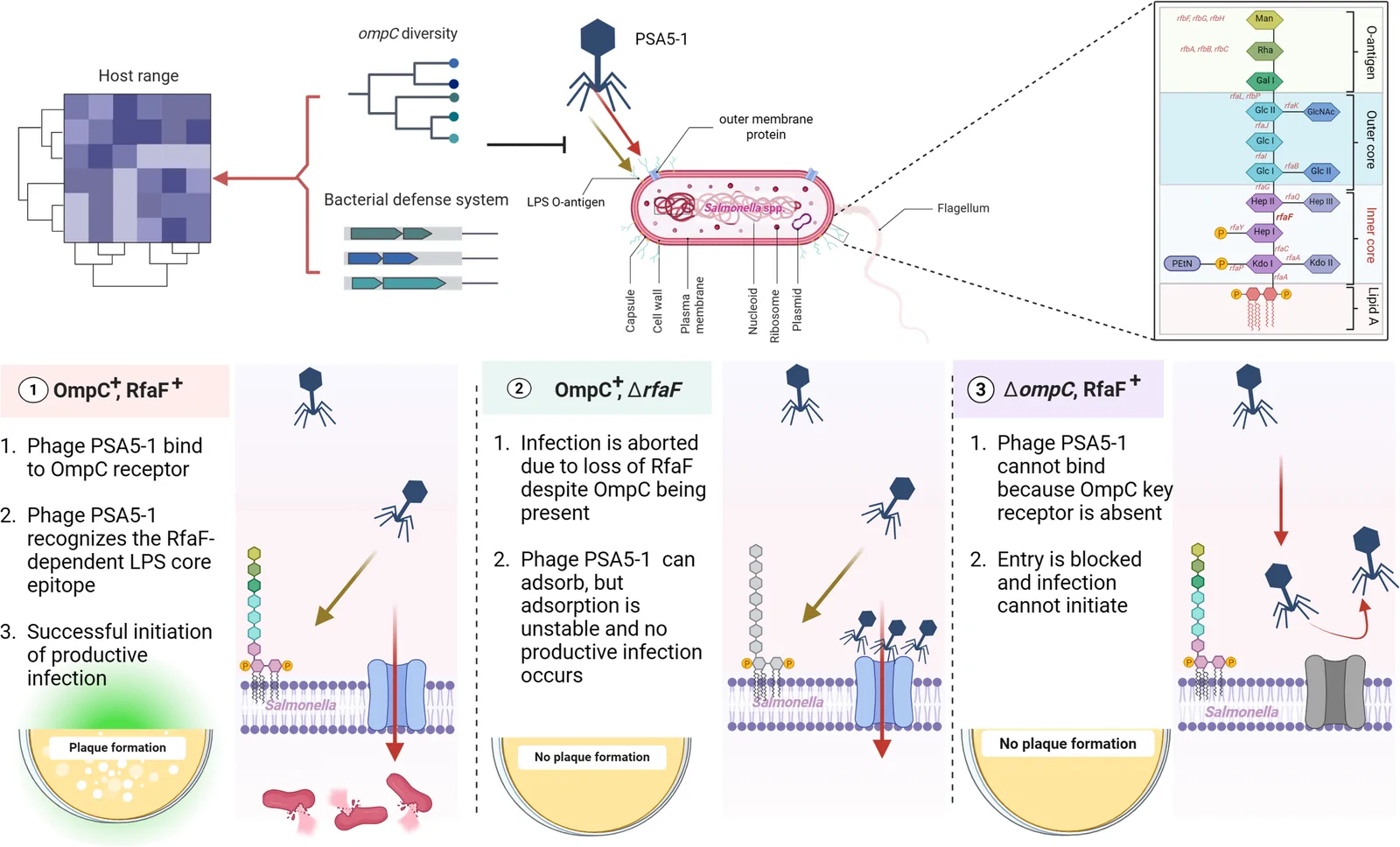
Multilayered host determinants governing PSA5-1 susceptibility in *Salmonella*. Schematic model illustrating how extracellular receptor architecture and intracellular immunity determine strain-specific susceptibility to the T4-like phage PSA5-1. Productive infection requires coordinated recognition of the OMP OmpC and an intact *rfaF*-dependent LPS inner core. OmpC^+^ RfaF^+^ strains support efficient adsorption, genome delivery, and plaque formation. OmpC^+^ Δ*rfaF* strains permit initial binding but fail to progress to productive infection due to disrupted LPS core structure. Δ*ompC* RfaF^+^ strains lack the essential receptor and are completely resistant to phage attachment. This reveals that OmpC microdiversity, LPS core integrity, intracellular defense systems, and prophage content define PSA5-1 host range and infection outcome in *Salmonella*.

In this study, we characterize PSA5-1, a lytic *Straboviridae* phage isolated from poultry feces and dissect the genetic and phenotypic determinants of host susceptibility across twelve *Salmonella* isolates. By integrating genome-wide host profiling with targeted mutagenesis and complementation, we show that PSA5-1 infection relies on a two-step receptor recognition process, with OmpC mediating binding and the *rfaF*-dependent LPS inner core, enabling productive infection and progeny release (Fig. 1). These findings show how OmpC receptor diversity, LPS O-antigen architecture, and intracellular defense and prophage landscapes jointly shape phage susceptibility and resistance, providing a mechanistic framework for developing evolution-aware, receptor-guided phage therapies to combat *Salmonella* across zoonotic reservoirs.

## RESULTS

### Isolation and characterization of the *Straboviridae* phage PSA5-1

**Fig 2:**
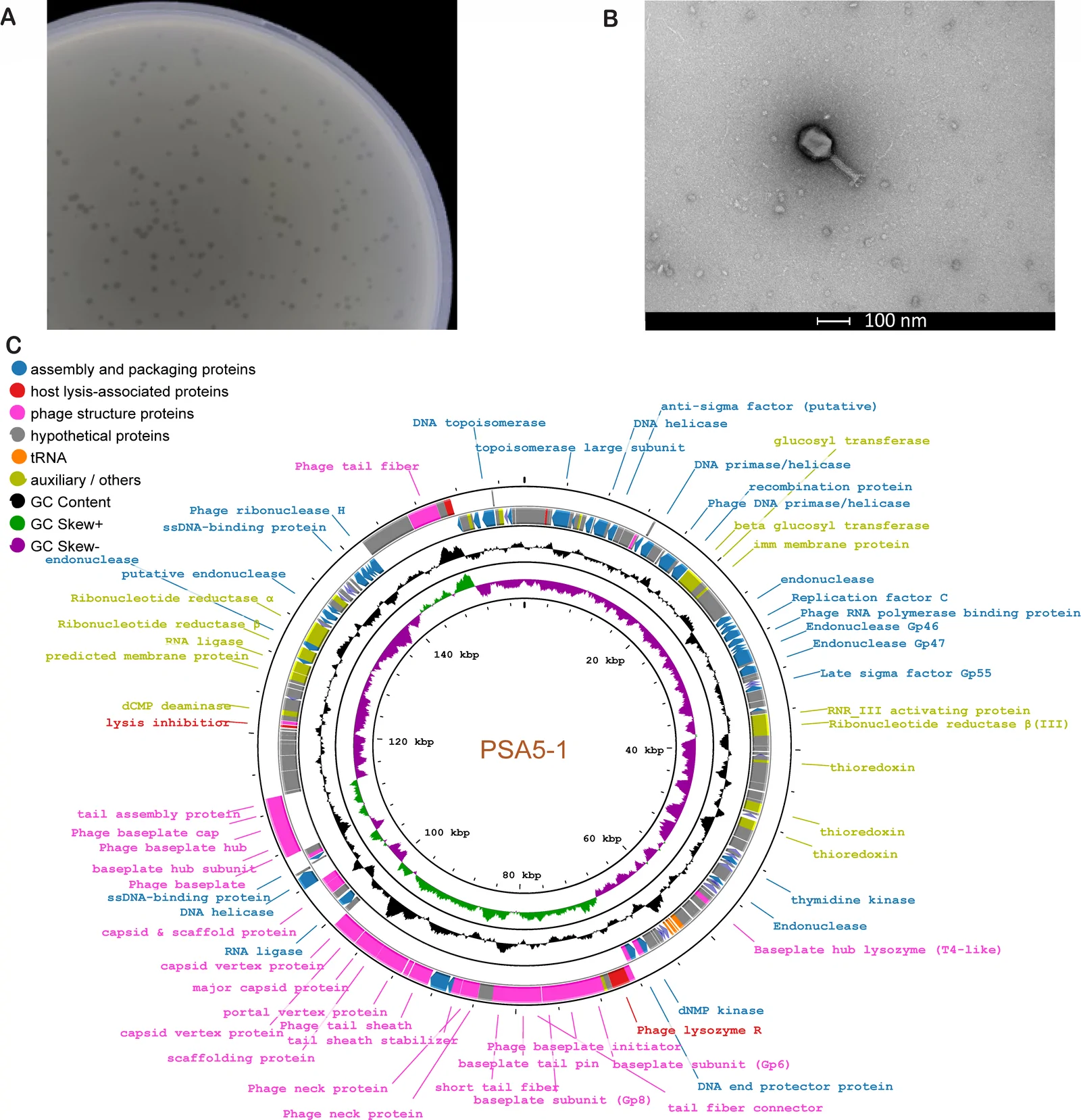
Morphological and genomic characterization of bacteriophage PSA5-1. (**A**) Plaque morphology of PSA5-1 on *S*. typhimurium S12 lawns. PSA5-1 forms clear, circular plaques (~1–2 mm in diameter), consistent with a strictly lytic lifestyle. (**B**) Transmission electron micrograph of PSA5-1 following negative staining, revealing an icosahedral capsid (~110 nm in length and ~ 75 nm in width) attached to a contractile tail (~115 nm in length), characteristic of T4-like myoviruses within the family Straboviridae. Scale bar, 100 nm. (**C**) Circular genome map of PSA5-1 generated using Proksee. The 158,395-bp genome (GC content, 36.97%) is organized into functional modules, including DNA replication and packaging (blue), host lysis (red), structural proteins (pink), auxiliary/anti-host functions (green), hypothetical proteins (gray), and tRNA genes (orange). The inner ring indicates GC content variation across the genome.

Phage PSA5-1 was isolated from poultry feces using *S. enterica* S12 as the host. PSA5-1 produced clear, transparent circular plaques, suggesting a strictly lytic lifestyle (Fig. 2A). Transmission electron microscopy revealed an icosahedral capsid (~110 nm in length, ~75 nm in width) attached to a contractile tail (~115 nm), characteristic of T4-like myoviruses within the family *Straboviridae* (Fig. 2B) (29). Agarose gel electrophoresis confirmed the quality of extracted PSA5-1 genomic DNA (Fig. S1). Whole-genome sequencing yielded a linear dsDNA genome of 158,395 bp with a GC content of 36.97%. CheckV confirmed 93.98% genome completeness and high quality. A total of 255 open reading frames (ORFs) along with 11 tRNA genes were predicted. The genome exhibited a modular organization typical of T4-like phages. Replication and nucleotide metabolism genes comprised 56 ORFs (22%), including DNA polymerase, helicase, primase, topoisomerase, ribonucleotide reductase, thymidylate synthase, thioredoxin, and DNA adenine methyltransferase. Transcriptional regulation accounted for 14 ORFs (5.5%), including sigma and anti-sigma factors. Morphogenesis and DNA packaging included 47 ORFs (18.4%), encoding terminase subunits, portal, capsid, tail sheath, tube, baseplate, and tail fiber proteins. The presence of gp37 (ORF236) and its chaperone gp38 (ORF237) confirms conserved homology with T4-like S16 tail fiber architecture involved in host recognition (Fig. S8A and B). The lysis module contained 5 ORFs (2%), including holin and endolysin (lysozyme R). An additional six ORFs (2.4%) encoded auxiliary metabolic and anti-host proteins, such as phospholipase and anti-restriction factors. The remaining 118 ORFs (46.3%) were annotated as hypothetical proteins, indicating substantial uncharacterized functional diversity (Fig. 2C). No antimicrobial resistance or bacterial virulence genes were detected, and BACPHLIP classified PSA5-1 as strictly lytic. Comparative genomic analyses against representative *Straboviridae* phages revealed that PSA5-1 is most closely related to phage Melville (99.62% identity; 99% coverage), followed by CF-SP2, STmL-198, and vB_SenM-AKM_NP4 (Table S1). Protein clustering and ANI analysis confirmed >95% identity with members of the *Gelderlandvirus* genus, while showing <75% identity to *Moonvirus* and *Karamvirus*, supporting taxonomic distinction (Fig. S2A and B). Phylogenetic reconstruction using conserved marker genes, including the large terminase subunit and major capsid protein, consistently placed PSA5-1 within the family *Straboviridae*, subfamily Tevenvirinae, clustering with T4-like Gelderlandvirus phages (Fig. S3 and S4).

### Comparative genomic profiling pinpoints key loci underlying phage susceptibility, resistance, and trade-offs

**Fig 3:**
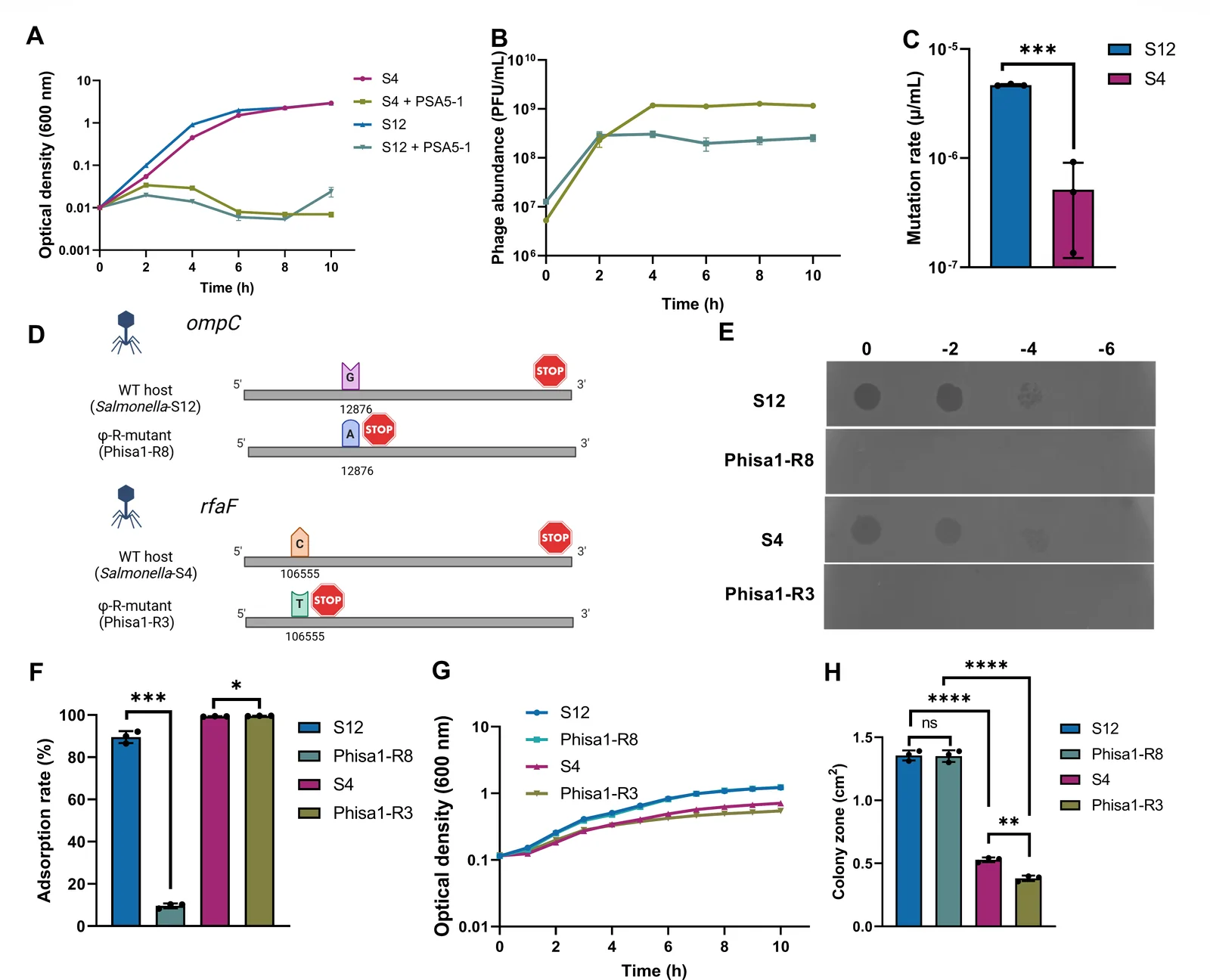
Genetic, adsorption, and fitness characterization of PSA5-1-resistant *Salmonella* mutants. (**A**) Growth curves of *Salmonella* strains S12 and S4 cultured in the presence or absence of PSA5-1. (**B**) Phage abundance over time following infection of S12 and S4. (**C**) Mutation rates associated with the emergence of PSA5-1-resistant mutants in S12 and S4. (**D**) Schematic representation of mutations affecting the OMP OmpC and the LPS inner-core biosynthesis enzyme RfaF in resistant mutants. (**E**) Spot assays showing PSA5-1 plaque formation on wild-type strains and corresponding resistant derivatives. (**F**) Phage adsorption efficiencies measured for wild-type and resistant strains. (**G**) Growth curves comparing wild-type strains and PSA5-1-resistant mutants in liquid culture. (**H**) Quantification of colony size on solid media for S4 and S12 wild-type and their resistant mutants (Phisa1-R8; Phisa1-R3). Bar graphs represent mean values with error bars, and statistical comparisons are indicated where applicable. *P < 0.05, **P < 0.01, ***P < 0.001; and ****P < 0.0001; ns, not significant.

To define how *Salmonella* resists infection by the T4-like phage PSA5-1, we focused on two representative hosts identified by initial screening: the highly susceptible strain S12 and the moderately susceptible strain S4 (Fig. S5). Although PSA5-1 strongly inhibited growth in both backgrounds, infection outcomes diverged markedly. S12 exhibited markedly suppressed bacterial growth but supported limited phage amplification, whereas S4 displayed delayed lysis yet produced substantially higher phage yields, indicating strain-specific constraints on productive replication (Fig. 3A and B). Resistance emerged at a significantly higher frequency in S12 than in S4, revealing distinct evolutionary trajectories (Fig. 3C). Whole-genome sequencing of eight stable S12-derived resistant mutants showed that resistance consistently arose from high-impact mutations in the OMP gene *ompC* or its regulatory two-component system EnvZ/OmpR, resulting in loss of porin expression, preventing productive infection (Fig. S6A). These mutants, exemplified by Phisa1-R8, were completely refractory to PSA5-1, establishing OmpC as the primary receptor required for efficient adsorption. In contrast, three resistant S4-derived mutants carried mutations in *rfaF*, encoding ADP-heptose heptosyltransferase II required for inner-core LPS assembly (Fig. 3D and E; Table S2). Despite retaining intact OmpC, these mutants, represented by Phisa1-R3, failed to support plaque formation (Fig. S6B), indicating that LPS core integrity is required for productive infection (Fig. 3E).

Adsorption assays resolved this mechanistic distinction. Loss of *ompC* in Phisa1-R8 severely reduced phage binding, whereas *rfaF* disruption in Phisa1-R3 had no detectable effect on adsorption, uncoupling attachment from productive infection. These results demonstrate that PSA5-1 requires both OmpC-mediated reversible binding and an intact *rfaF*-dependent LPS inner core for irreversible attachment, genome delivery, and productive infection (Fig. 3F). Growth curve analyses revealed contrasting fitness consequences of these resistance strategies. The S12-derived *ompC* mutant Phisa1-R8 grew similarly to wild-type S12 during early logarithmic phase but exhibited a modest growth delay (*P* < 0.05) at later time points, consistent with the physiological cost of porin loss. In contrast, the S4-derived *rfaF* mutant Phisa1-R3 displayed significantly reduced growth in liquid culture compared with wild-type S4, particularly after mid-log phase (*P* < 0.0001) (Fig. 3G), and formed significantly smaller colonies on solid media, whereas the *ompC* mutant Phisa1-R8 showed no detectable colony-size difference relative to wild-type S12 (Fig. 3H). Collectively, these results highlight that *ompC*-mediated resistance is largely fitness-neutral at the colony level, whereas *rfaF*-mediated resistance imposes measurable trade-offs, highlighting how alternative receptor-centered escape routes to PSA5-1 resistance generate divergent physiological outcomes.

### OmpC-mediated binding and LPS core integrity regulate PSA5-1 infection

**Fig 4:**
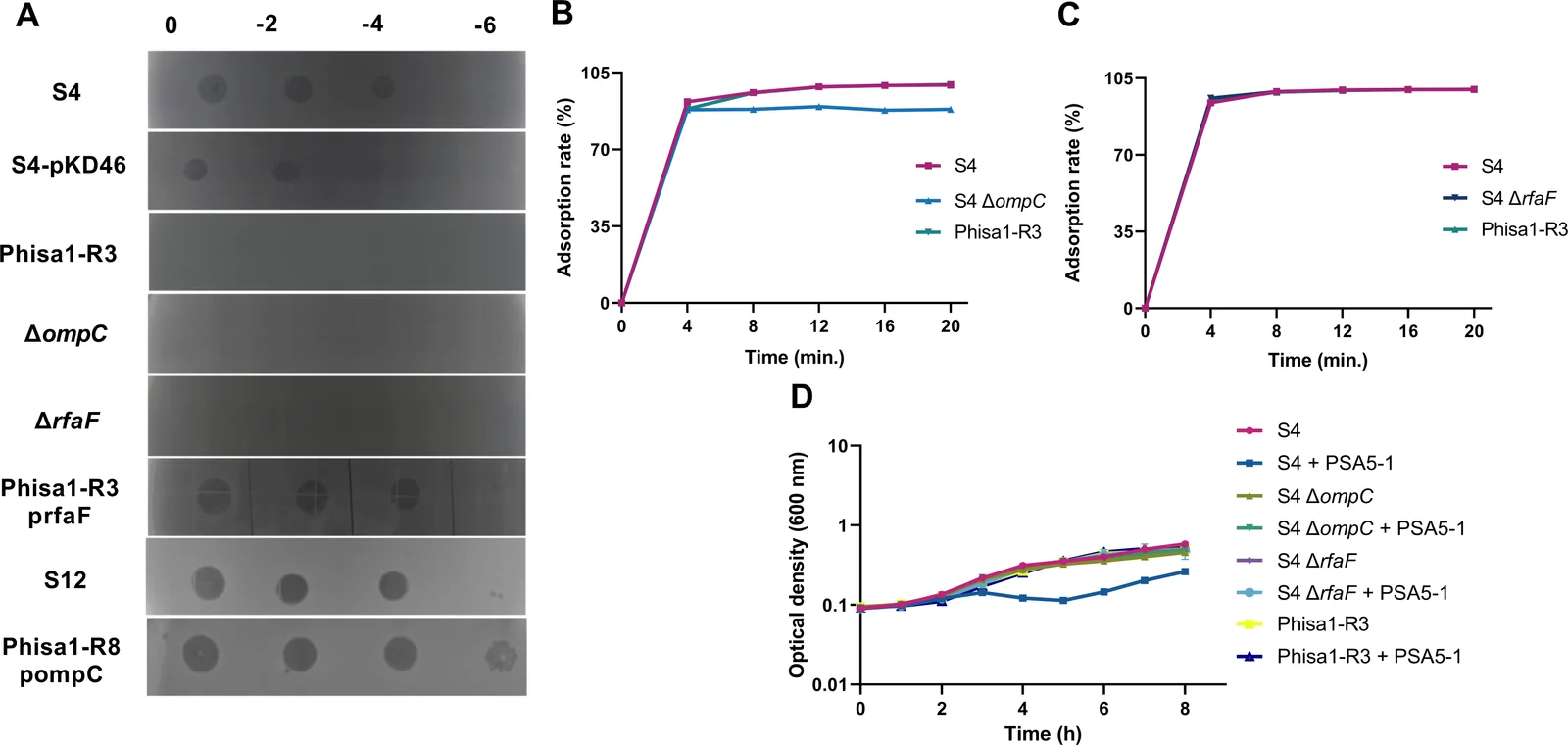
*ompC* and *rfaF* mutations alter PSA5-1 adsorption. (**A**) Spot assays showing PSA5-1 plaque formation on wild-type, plasmid control, deletion mutants, and complemented strains at serial dilutions (10⁰–10⁻⁶). (**B and C**) Time-resolved adsorption assays showing the percentage of PSA5-1 adsorption over 20 min for wild-type S4, and its receptor mutants (Phisa1-R3, Δ*ompC*, Δ*rfaF*). (**D**) Growth curves of wild-type S4, receptor mutant strains following challenge with PSA5-1.

To define the receptor requirements of PSA5-1, we generated in-frame disruptions of *ompC* and *rfaF* in the S4 background and compared these engineered mutants with wild-type S4 and spontaneous phage-resistant derivatives arising from different host backgrounds (Phisa1-R3 from S4 and Phisa1-R8 from S12). Disruption of either *ompC* or *rfaF* completely abolished plaque formation in S4, demonstrating that both OmpC and an intact *rfaF*-dependent LPS inner core are essential for productive infection. Genetic complementation from the arabinose-inducible plasmid pHB20TG fully restored susceptibility in a background-specific manner: expression of wild-type *rfaF* rescued the S4-derived mutant Phisa1-R3, while expression of *ompC* restored lysis in the S12-derived mutant Phisa1-R8, confirming the specific requirement of each locus for PSA5-1 susceptibility (Fig. 4A).

Adsorption assays revealed a clear mechanistic distinction between the two receptors. Loss of OmpC caused a pronounced defect in early phage binding in S4 Δ*ompC*, with substantially higher levels of unadsorbed PSA5-1 and lower adsorption efficiency compared with the OmpC-positive wild-type S4 strain (*P* < 0.001), establishing OmpC as the essential primary adsorption receptor. In contrast, the *rfaF* mutant Phisa1-R3 exhibited an adsorption rate comparable to wild-type S4 during early time points, indicating that *rfaF* disruption does not impair initial attachment (Fig. 4B). Consistently, the engineered S4 Δ*rfaF* strain also showed an adsorption profile similar to wild-type S4, reaching near-complete adsorption within 12–20 min. Comparisons between S4 Δ*rfaF* and Phisa1-R3 further showed that *rfaF*-dependent LPS core mutations altered adsorption kinetics without affecting the final extent of binding, consistent with a block at a post-attachment step required for irreversible attachment or downstream infection (Fig. 4C). This hierarchical receptor requirement was conserved for the related *Straboviridae* (T4-like) phages. We next evaluated how these receptor alterations shape bacterial susceptibility to PSA5-1 in liquid culture. Deletion of *ompC* or *rfaF* conferred complete resistance in liquid culture, supporting a S16-like model in which either gene is indispensable for productive infection (Fig. 4D). Together, these results define a hierarchical two-step receptor model in which OmpC functions as the primary adsorption receptor for PSA5-1, while *rfaF*-dependent LPS core integrity is dispensable for adsorption and governs post-attachment steps required for downstream productive infection.

### *rfaF*-mediated LPS core remodeling drives biofilm formation and surface-associated motility

**Fig 5:**
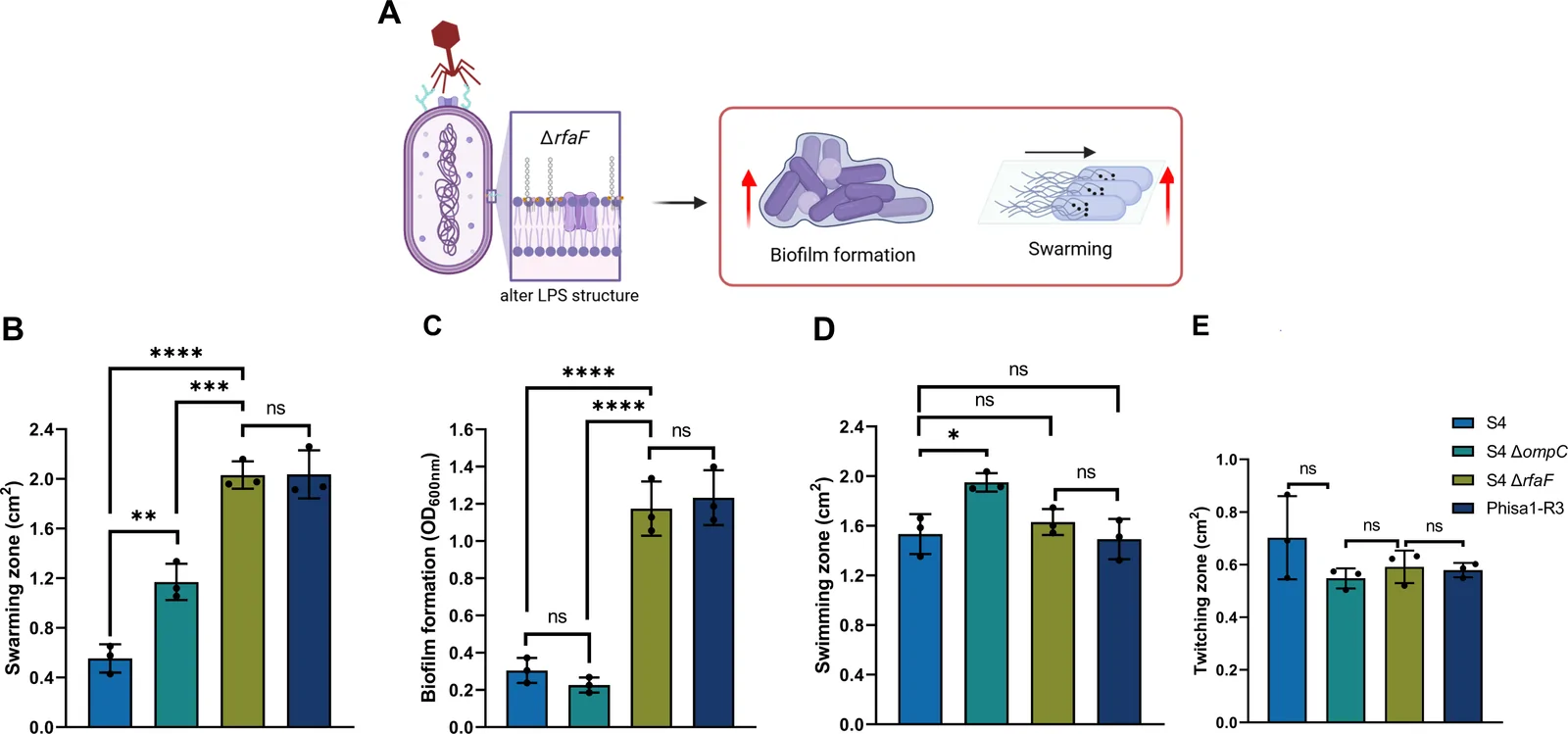
Effects of *ompC* and *rfaF* mutations on surface-associated phenotypes in *Salmonella* S4. (**A**) Schematic summary illustrating surface-associated phenotypes examined in *Salmonella* S4 backgrounds following disruption of *ompC* or *rfaF*. (**B**) Swarming motility of S4 wild-type, Δ*ompC*, Δ*rfaF*, and Phisa1-R3 strains measured on soft agar. (**C**) Quantification of biofilm formation in S4 wild-type and derivative strains. (**D**) Swimming motility of S4 wild-type, Δ*ompC*, Δ*rfaF*, and Phisa1-R3 strains measured on soft agar for 72 h. (**E**) Twitching motility assay comparing S4 wild-type and its mutant derivatives. Bar graphs represent mean values with error bars, and statistical comparisons are indicated where applicable. *P < 0.05, **P < 0.01, ***P < 0.001; and ****P < 0.0001; ns, not significant.

To evaluate fitness trade-offs associated with PSA5-1 resistance, we examined surface-associated behaviors in *Salmonella* S4 and its receptor-mutant derivatives (Fig. 5A). In the S4 background, resistance was accompanied by pronounced, receptor-specific phenotypic shifts. Disruption of *rfaF* produced a striking hyper-swarming phenotype (Fig. 5B) and significantly enhanced biofilm formation (*P* < 0.0001), which was observed both in the engineered Δ*rfaF* mutant and the spontaneous resistant mutant Phisa1-R3, indicating that LPS inner-core remodeling promotes collective surface-associated phenotypes. In contrast, loss of *ompC* caused only modest changes in swarming (*P* = 0.0036) and did not significantly alter biofilm formation (*P* = 0.82), suggesting limited physiological impact of porin loss on multicellular behavior in S4 (Fig. 5C). Flagellar-driven swimming remained largely intact in *rfaF* mutants (Fig. 5D), and pili-driven twitching motility was unchanged across all S4 derivatives (*P* = 0.2336) (Fig. 5E), indicating that LPS-core disruption selectively rewires surface behaviors without broadly impairing motility systems. Consistent with this, these results demonstrate that *rfaF*- and *ompC*-mediated resistance impose fundamentally different physiological outcomes: LPS core remodeling in S4 drives hyper-swarming and hyper-biofilm formation, whereas porin loss in S4 favors swarming motility.

### PSA5-1 exhibits a broad but structured host range across poultry-derived *Salmonella* isolates

**Fig 6:**
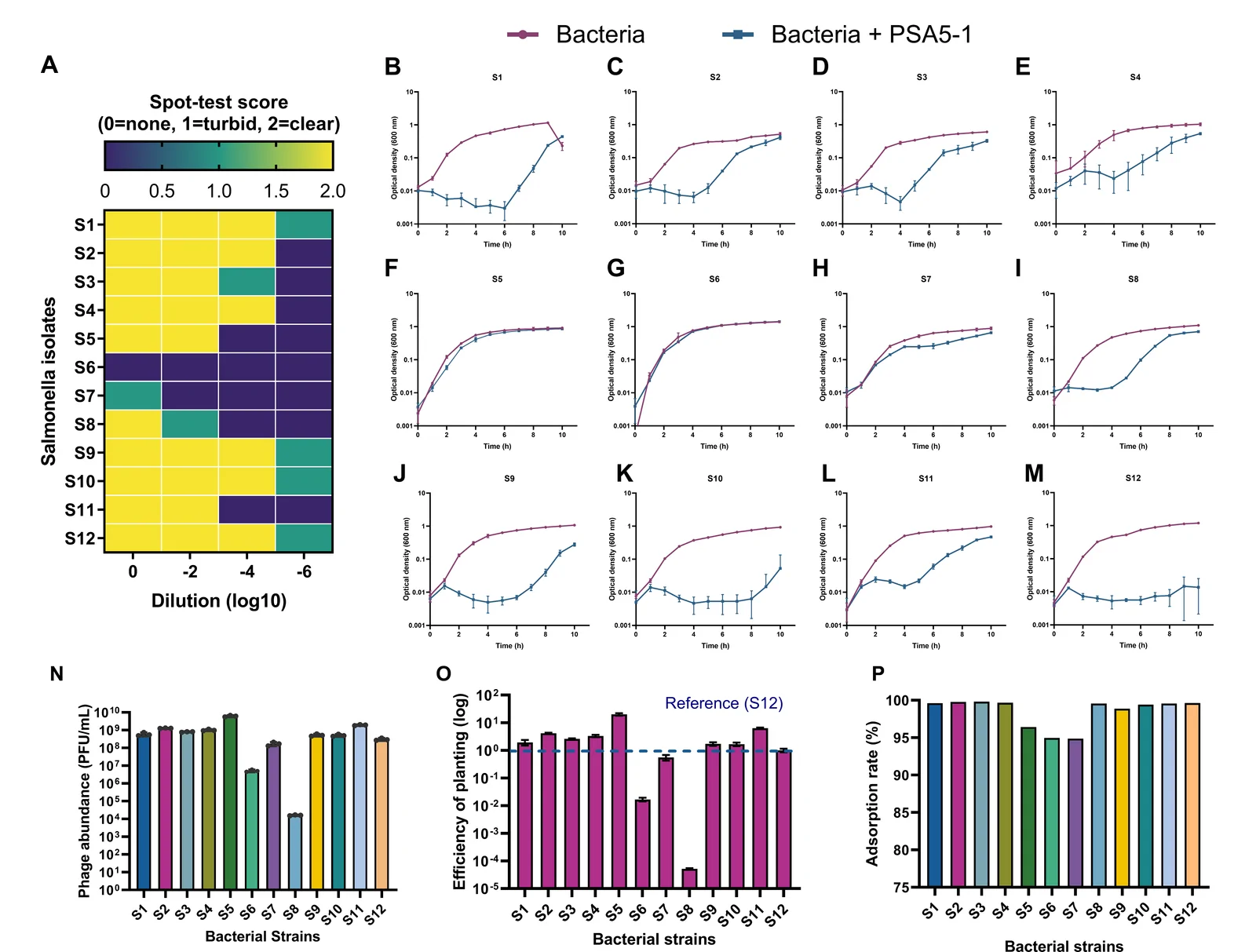
Host-range profiling and infection dynamics of PSA5-1 across twelve *Salmonella* isolates. (**A**) Spot-test heatmap showing PSA5-1 lytic activity against twelve *S. enterica* isolates at dilutions ranging from 10⁰–10⁻⁶ (PFU/spot). Color scale indicates clearing scores (0, no clearing; 1, turbid; 2, clear). (**B–M**) Growth curves of each isolate over 10 h in the absence (blue) or presence (green) of PSA5-1. (**N**) Phage progeny production measured at 4 h post-infection for each isolate. (**O**) Efficiency-of-plating (EOP) calculated relative to the reference host S12 (dashed blue line). (**P**) Percentage of PSA5-1 adsorption to each isolate measured during early infection. Data are shown as mean values with error bars where applicable.

To define the host range and productive capacity of PSA5-1, we examined phage infection across 12 poultry-derived *S. enterica* isolates. Consistent with its dependence on OmpC, PSA5-1 infected most strains, with only S6 showing near-complete resistance. Strain S7 produced turbid plaques and showed <1 log reduction at 10^8^ PFU/mL, consistent with inefficient infection or lysis from without rather than productive replication; receptor recognition and adsorption remained efficient, suggesting that the infection is restricted after adsorption (Fig. 6A). Infection assays revealed marked strain-specific differences in productive infection. Nine isolates supported rapid and sustained growth suppression (*P* < 0.0001), whereas S5 and S6 showed minimal inhibition (Fig. 6F and G). Among susceptible strains, the strongest inhibition occurred in S9, S10, and S12 (*P* < 0.0001) (Fig. 6J, K and M), followed by moderate inhibition in S3, S4, S7, S8 (Fig. 6D, E, H and I), and S11 (*P* < 0.001–0.0001) (Fig. 6L), while S2 displayed a weaker but significant effect (*P* < 0.01–0.001) (Fig. 6C). Strain S1 showed early inhibition but partial regrowth by 10 h (*P* = 0.0003), suggesting resistance emergence (Fig. 6B). Maximal lysis occurred between 6 and 8 h, coinciding with peak phage propagation. Overall, the extent of growth inhibition varied substantially, indicating that adsorption alone was insufficient to predict infection outcome. Direct quantification of phage progeny revealed pronounced host-dependent differences in productivity. Strains S4, S5, S10, and S11 supported the highest phage yields (~10⁹–10^10^ PFU/mL), and likely harbored highly compatible receptors that supported robust phage amplification. Several strains, including S1, S2, S3, S9, and S12 produced intermediate titers (~10⁸–10⁹ PFU/mL), while S7 showed slightly lower yields (~10⁸ PFU/mL). In contrast, S6 exhibited markedly minimal progeny (~10⁶ PFU/mL), mirroring its partial growth inhibition. Notably, S8 generated extremely low phage yield (~10^4^ PFU/mL), indicating near-complete inhibition of productive infection despite efficient adsorption (Fig. 6N). Efficiency of plating (EOP) analysis mirrored these trends. Using S12 as the reference host, strains S5 and S11 exhibited the highest EOP, indicating highly efficient plaque formation and productive infection. Strains S2, S3, S4, S9, and S10 showed moderate to high EOP, consistent with effective infections. S1 displayed a modest EOP, while S7 showed reduced efficiency, consistent with partial restriction. In contrast, S6 and S8 showed an extremely low EOP, indicating near-complete resistance phenotypes. Notably, S5 exhibited high apparent sensitivity, despite weak growth inhibition. Because EOP reflects plaque formation on bacterial lawns and does not necessarily reflect phage amplification in liquid culture, discrepancies between plaque formation and productive infection can arise due to differences in host physiology, receptor density, adsorption kinetics, or burst size (Fig. 6O). Adsorption assays further clarified these patterns. PSA5-1 bound efficiently to most isolates (>94%), including several strains with poor replication, demonstrating that receptor recognition permits attachment but does not guarantee productive infection. The highest adsorption rates were observed in S2 and S3 (99.7%–99.8%), while slightly reduced adsorption was seen in S5, S6, and S7 (94%–96%). Notably, S8 also exhibited high adsorption despite minimal EOP and phage production, confirming a strong post-adsorption restriction phenotype (Fig. 6P).

### Multilayered host determinants shape strain-specific susceptibility to phage PSA5-1

**Fig 7:**
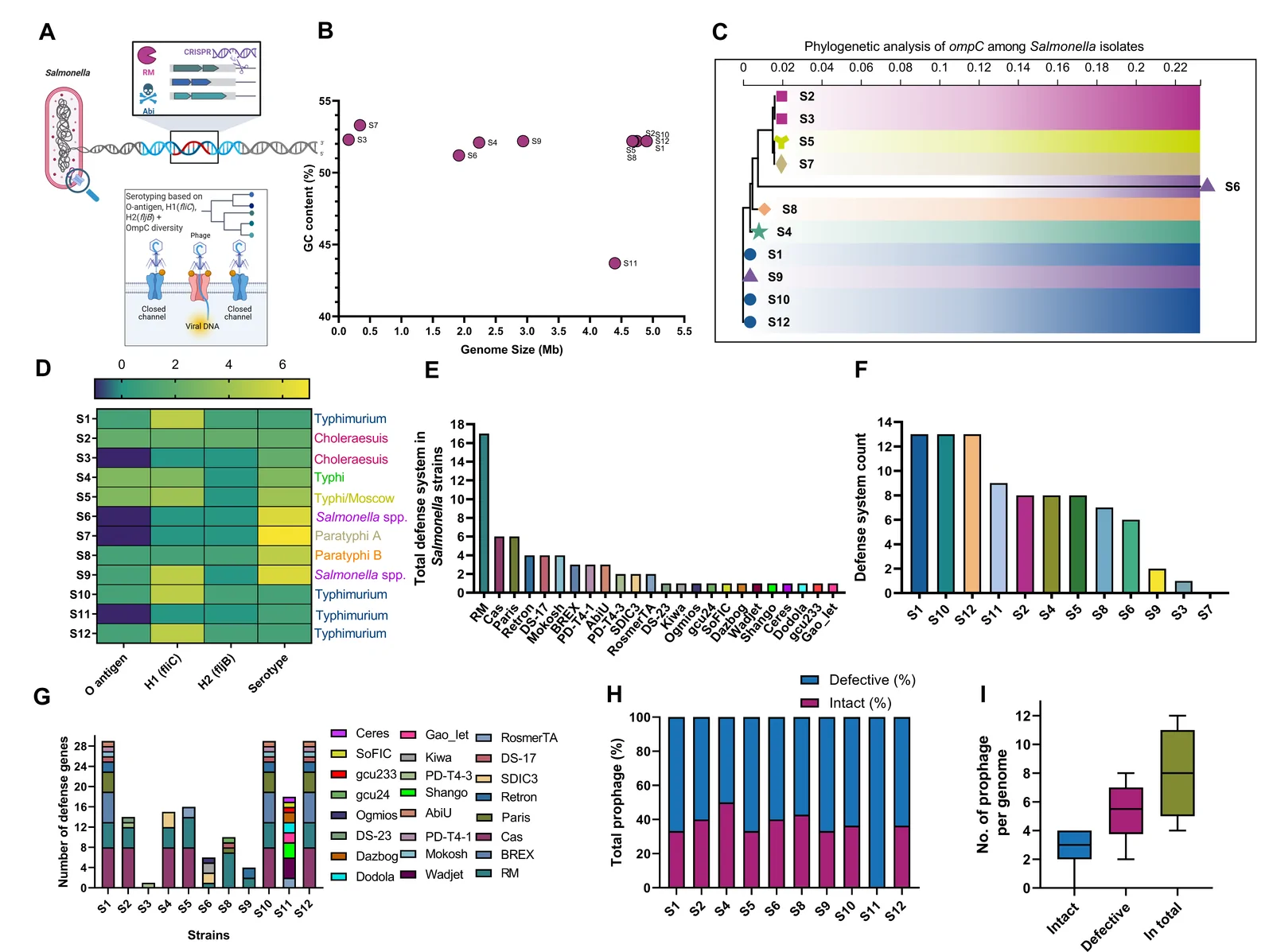
Comparative genomic, receptors, serotypic, and defense variation across twelve *Salmonella* isolates. (**A**) Schematic overview of genomic and surface features analyzed across *Salmonella* isolates, including antiviral defense systems, O-antigen structure, and porin-mediated phage adsorption via OmpC. (**B**) Genome size and GC content distribution for each isolate. (**C**) Phylogenetic reconstruction of OmpC protein sequences illustrating sequence conservation and divergence among isolates, strain S11 represents an outlier; lacking functional *ompC* yet displaying high susceptibility to PSA5-1, suggesting the presence of an alternative receptor or compensatory mechanism. (**D**) Heatmap summarizing O-antigen type (O = 4, 7, 9, −1), H1 antigen (*fliC* = i, c, d, gmq, **B**), H2 antigen (*fljB* = 1, 2/1, 5) flagellar markers, and serovar assignments, while –1 or 0 indicates absence or undetermined prediction (Table S7). (**E**) Total number of identified antiviral defense systems per isolate, grouped by defense class. (**F**) Defense system counts across individual strains. (**G**) Composition of defense genes in each isolate, shown as stacked bars representing distinct defense families. (**H**) Proportion of intact and defective prophages in each genome. (**I**) Boxplot summarizing intact, defective, and total prophage counts across the isolate panel.

Phage susceptibility and fitness are often shaped by a multilayered interplay between extracellular receptor architecture, intracellular immune defenses, and lineage-specific genomic features. The broad yet nonuniform host range of PSA5-1 observed across twelve *Salmonella* isolates supports the hypothesis that infection outcomes are governed by the combined effects of OmpC receptor compatibility, LPS O-antigen architecture, defense-system burden, and prophage content, rather than by receptor presence alone (Fig. 7A). Comparative genomic analysis revealed substantial heterogeneity among the strain panel (S1 to S12). Genome sizes varied widely, while GC content remained conserved (51.2%–53.3%), reflecting substantial evolutionary diversity within this panel of strains (Fig. 7B). Understanding the amino acid-level determinants of receptor recognition, in the hypervariable extracellular loops of receptors such as OmpC, is essential for predicting phage host range and selecting effective therapeutic phages. Analysis of the host receptor OmpC locus revealed strong overall conservation consistent with its essential physiological role, including uniform length (378 aa), predicted β-barrel topology, membrane localization, and physicochemical properties. However, fine-scale divergence within extracellular loops, key determinants of phage binding, correlated closely with PSA5-1 susceptibility and related phages such as S16 (30). Most isolates (S1, S4, S8–S10, S12) carried OmpC variants with 99%–100% identity to the PSA5-1 reference host S12, forming a tightly clustered phylogenetic group. A second set of isolates (S2, S3, S5, S7) exhibited ~98% identity, indicating only minor substitutions that are unlikely to disrupt overall porin architecture but may subtly affect receptor epitopes. In contrast, strain S6 encoded a markedly divergent OmpC, sharing only 81.5% identity with S12 and possessing a slightly shortened CDS (376 aa) (Table S3). Phylogenetically, S6 formed a distinct long branch, separated by >0.19 evolutionary distance units from all other isolates and displayed near-complete resistance. Our results identify *ompC* as the main receptor for PSA5-1 across many tested strains, the high susceptibility of strain S11, which lacks a functional *ompC*, suggests a secondary or “bypass” entry mechanism. This does not invalidate the essentiality of *ompC* in a general context but rather highlights the opportunistic binding strategies common to T4-like phages (Fig. 7C; Fig. S7). This degree of amino acid divergence strongly suggests remodeling of key extracellular loops (L2–L5)—the same regions in *Enterobacteriaceae* known to dictate specificity for outer membrane-targeting phages such as *Straboviridae* (T4-like) viruses (30–32). These loop variants occur not only across *Escherichia coli* populations but also in related pathogenic species (e.g., *Mannheimia haemolytica*, *M. glucosida*, *Pasteurella trehalosi*) (33, 34). These data suggest that subtle amino acid substitutions within otherwise conserved OmpC proteins can decisively alter PSA5-1 infection efficiency.

Moreover, surface antigens, including the O-antigen and flagellar determinants (H1 *fliC* and H2 *fljB*), stratified the twelve *Salmonella* isolates into multiple serovars, including Typhimurium (S1, S10, S11, S12), Choleraesuis (S2, S3), Typhi (S4), Typhi/Moscow (S5), Paratyphi A (S7), Paratyphi B (S8), and *Salmonella* spp. (S6, S9), underscoring the extensive antigenic landscape encountered by PSA5-1. O-antigen composition ranged from smooth LPS types (O:4 in S1, S8, S9, S10, and S12; O:9 in S4 and S5; and O:7 in S2) to rough phenotypes lacking identifiable O-antigen loci in several isolates (S3, S6, S7, S11), a variation expected to influence receptor accessibility and steric masking (Fig. 7D).

Next, defense-system profiling identified 24 distinct antiviral families with highly uneven distribution across isolates. RM systems were the most abundant, followed by CRISPR-Cas, Paris, Retron, DS-17, and Mokosh. Several systems—including BREX, PD-T4-type modules, AbiU, and RosmerTA—occurred sporadically, whereas rare systems, such as Kiwa, Wadjet, Ogmios, Dazbog, and Ceres, were detected only once, indicating isolate-specific acquisition events (Fig. 7E; Table S4). Defense-rich strains, such as S1, S10, and S12, encoded up to 13 systems, forming dense multilayered defense islands, whereas S11, S2, S4, and S5 harbored intermediate defenses (8–9 systems). S8 and S6 carried only seven and six systems, respectively, while S9 encoded just two systems. S3 carried a single identifiable defense gene, and S7 lacked any detectable system and possessed an OmpC receptor highly similar to other strains; S7 displayed strong resistance to PSA5-1. The phage adsorbed efficiently to S7, indicating that receptor recognition remained intact. However, productive infection was severely limited, as evidenced by minimal growth inhibition and low phage production. This pattern indicates a post-adsorption restriction, where phage attachment occurs but subsequent steps of infection are impaired. One possible explanation is cell envelope variation, as S7 exhibits a rough LPS phenotype lacking O-antigen, which may affect LPS core accessibility required for efficient PSA5-1 engagement or DNA injection (Fig. 7F). At the gene level, high-defense strains (S1, S10, S12) encoded multilayered architectures, including abundant Cas, RM, and BREX systems. Intermediate strains (S2, S4, S5) maintained strong RM-Cas backbones but lacked BREX or Paris systems. Defense-poor isolates (S3, S7, S11) either lacked canonical systems or relied exclusively on mobile toxin–antitoxin modules (Fig. 7G; Table S5). Strains with extensive defense islands frequently restricted PSA5-1 replication despite efficient adsorption, indicating that intracellular immunity constitutes a dominant secondary checkpoint following entry.

Prophage landscapes added an additional layer of heterogeneity. Most strains carried 4 to 12 prophages, predominantly defective elements. Intact prophages represented 33% to 43% of total prophage content in most isolates (S1, S2, S5, S6, S8, S9, S10, S12), whereas S11 contained exclusively defective remnants, with no intact prophages, suggesting different evolutionary histories of phage exposure and defense activation. Only S4 contained approximately 50% intact prophage regions, along with additional questionable and incomplete prophage elements, indicating a relatively balanced prophage composition compared with the other analyzed strains. Intact prophages can influence bacterial susceptibility to lytic phages through mechanisms such as superinfection exclusion or regulatory interference, potentially affecting phage adsorption or replication and contributing to the phage–host interaction patterns observed in a filamentous Pf bacteriophage system (35) (Fig. 7H and I; Table S6). Strains enriched in intact prophages tended to show reduced PSA5-1 productivity, consistent with prophage-encoded superinfection exclusion or regulatory interference, whereas strains dominated by defective remnants were more permissive to lytic replication. Our data suggest a potential role for prophage-mediated superinfection exclusion, though further molecular validation is required to definitively link these specific prophage contents to the observed resistance. Collectively, these results establish that PSA5-1 susceptibility across *Salmonella* is a composite trait shaped by coordinated extracellular receptor compatibility and intracellular restriction layers. OmpC microdiversity, O-antigen context, defense-system load, and prophage composition together define strain-specific infection outcomes, underscoring the complexity of phage–host interactions that must be considered when predicting host range and designing effective phage-based interventions.

## DISCUSSION

The rapid emergence of MDR *Salmonella spp*. has renewed interest in bacteriophage therapy as a precision alternative to antibiotics (36, 37). Phage infection is initiated by the specific interaction between phage-encoded RBPs and exposed bacterial surface structures, making receptor identification a critical first step in elucidating phage–host interactions. These receptors commonly include OMPs, LPS components, or surface polysaccharides, and productive infection often depends on the coordinated engagement of multiple receptors during reversible and irreversible adsorption stages. Dynamic modulation of these receptors is a key adaptive strategy enabling survival across heterogeneous ecological niches under intense viral predation. In Gram-negative pathogens, phage resistance frequently arises through alterations in receptor structure or regulation, often accompanied by remodeling of envelope architecture. In *Salmonella*, structural variation in OMPs, such as OmpC, and in the LPS core represents a major barrier to cross-serovar infectivity and drives receptor-mediated resistance evolution. Beyond surface specificity, *Salmonella* genomes encode dynamic defense “hotspots” that coordinate multilayered intracellular immunity against phage attack. Despite the well-established physiological roles of porins in osmoregulation and solute transport, how these proteins function as phage-accessible receptors, and how their activity depends on accessory envelope components, remains poorly defined. Here, we investigate the mechanistic basis of phage–host interactions using PSA5-1, a lytic T4-like phage infecting *Salmonella* isolates. We demonstrate that PSA5-1 infection follows a sequential receptor framework. Resistance arises through distinct receptor-centered pathways that impose divergent fitness and phenotypic trade-offs, revealing how receptor architecture, envelope integrity, and intracellular defenses collectively shape phage susceptibility in *Salmonella*.

Phage infection initiates through specific interactions between phage-encoded RBPs and exposed host surface receptors, making receptor identification central to understanding phage–host interactions and resistance evolution. Resistance can arise through extracellular mechanisms, such as mutations in surface receptors, including *ompC* and O-antigen, or through intracellular defenses that block subsequent infection stages. To dissect these processes for the strictly lytic *Straboviridae* phage PSA5-1, we focused on two strains—S12 and S4—that were both susceptible to PSA5-1 yet exhibited markedly different phage production and resistance trajectories. In the highly susceptible S12 background, resistance emerged at a markedly higher frequency and was consistently associated with high-impact mutations in *ompC*. In contrast, S4 yielded fewer resistant clones while supporting substantially higher phage amplification. Correspondingly, adsorption assays showed that the loss of *ompC* function in Phisa1-R8 reduced phage adsorption in S12 to near-background levels and eliminated plaque formation, establishing OmpC function as the adsorption receptor for PSA5-1. This finding aligns with extensive evidence that tailed *Straboviridae* phages commonly exploit the specific extracellular loops of outer membrane protein receptors and accounts for how their diversity affects phage binding and host range (33).

In contrast to S12, resistance in the moderately susceptible S4 strain followed a highly constrained evolutionary route, with all resistant mutants carrying nonsense mutations in *rfaF*, which is required for LPS inner-core assembly. Notably, disruption of *rfaF* did not impair initial phage attachment, as adsorption efficiencies in Phisa1-R3 and S4 Δ*rfaF* were indistinguishable from wild-type, indicating that OmpC-mediated binding remained intact. However, despite efficient adsorption, *rfaF* mutants completely failed to form plaques or support phage progeny release, demonstrating that the LPS core is required for productive infection and likely contributes to irreversible adsorption and downstream infection steps. This uncoupling of adsorption from productive infection mirrors classical T4-like phage infection models, in which initial binding to an outer-membrane protein is followed by engagement of LPS structures that trigger irreversible commitment and DNA ejection. Studies of bacteriophages T4, T2, and T6 have established a conserved two-step mechanism whereby LTFs mediate initial, reversible attachment, followed by irreversible engagement of LPS by STFs, inducing baseplate rearrangement, tail sheath contraction, and genome injection (22). In a previous study in *E. coli* K-12, T4 phage was shown to utilize both OmpC and LPS as receptors, with their relative importance dictated by LPS architecture: OmpC supports adsorption when the LPS core is truncated, whereas in OmpC-deficient strains adsorption relies exclusively on specific outer-core LPS structures. Collectively, these observations demonstrate that T4-like phages can exploit alternative adsorption routes depending on host envelope composition, with LTFs engaging LPS and lateral tail fiber surfaces interacting with OmpC (24). In contrast, some tailed phages, such as Kuttervirus GSP004 and the podovirus P22, use tail spike proteins to recognize a single receptor, typically the O-antigen (20). Notably, as a member of the *Straboviridae*, PSA5-1 conforms to this sequential multistep recognition paradigm but instead employs the general porin OmpC in conjunction with additional surface structures, with OmpC serving as the essential reversible receptor for adsorption, whereas a *rfaF-*dependent LPS inner core functions as a secondary determinant required for irreversible attachment and productive infection (33). This hierarchical receptor requirement mirrors classical phage S16 (T4-like) infection models, in which initial reversible binding to an outer-membrane protein is followed by engagement of LPS structures that trigger irreversible commitment and DNA ejection (13). Our data support a model in which OmpC functions as the receptor for reversible adsorption, while a *rfaF*-dependent LPS inner core serves as a secondary receptor required for irreversible attachment and downstream productive infection. This framework explains why *rfaF* mutants retain near-wild-type adsorption yet fail to form plaques, whereas loss of *ompC* abolishes adsorption entirely. Complementation experiments fully corroborate this model: restoration of *ompC* in Phisa1-R8 recovered susceptibility and plaque formation, while complementation of *rfaF* in Phisa1-R3 also restored plaque formation and phage replication. Multiple sequence alignment analysis of distal tail fiber proteins further supports this mechanism, as PSA5-1 gp37 and gp38 are highly similar to those of *Salmonella* phage S16 (76.4% and 89.9% identity, respectively) but divergent from T4 (28.4% and 15% identity) (Fig. S8A and B), suggesting an S16-like gp37–gp38 adhesin architecture where gp38 functions as a permanent distal adhesion rather than a transient assembly chaperone as in T4 phage (30, 38). Together, these findings demonstrate that PSA5-1 infection depends on coordinated recognition of both a protein receptor and a structurally intact LPS core, highlighting how multilayered receptor requirements shape host range and resistance evolution in *Salmonella*.

Surface modifications frequently perturb core membrane functions, including envelope integrity, motility, and adhesion, and often incur measurable fitness costs. Consistent with this principle, the resistance mechanisms observed here imposed distinct physiological trade-offs (39). Loss of OmpC function in the bacterial mutant Phisa1-R8 and S4 Δ*ompC* was largely fitness-neutral at the colony level, consistent with functional redundancy among OMPs. This observation aligns with previous studies in *E. coli*, where disruption of individual porins (*ompC* or *ompF*) had minimal effects on growth, reflecting compensatory permeability through alternative porins (40). In addition to the largely fitness-neutral OmpC pathway, *rfaF*-mediated resistance imposed a clear physiological cost, manifested as reduced colony size and growth in both Phisa1-R3 and S4 Δ*rfaF* despite preserved planktonic growth. These contrasting outcomes illustrate how receptor-centered resistance strategies differentially reshape host physiology and evolutionary trajectories under strong phage-imposed pressure. While disruption of *ompC* completely abolished PSA5-1 adsorption and infection with minimal impact on growth, it impaired biofilm formation without affecting twitching motility, consistent with the central structural and regulatory roles of porins in envelope homeostasis. OmpC is embedded within global regulatory networks, including the EnvZ/OmpR two-component system and sRNA-mediated control, such that its loss rewires the expression of alternative porins, curli fibers, and exopolysaccharides that collectively govern multicellular behavior. Similar phenotypes have been reported in avian pathogenic *E. coli*, where EnvZ/OmpR and MicC–Hfq-dependent repression of *ompC* coordinately modulate biofilm- and stress-associated genes, shifting the balance from stable biofilm formation toward increased surface motility (41, 42).

By contrast, *rfaF* mutants, lacking proper heptosylation of the LPS inner core and consequently producing deep-rough LPS with perturbed outer-membrane architecture (43), retained efficient phage adsorption yet completely blocked productive infection, while exhibiting markedly enhanced biofilm formation and swarming motility. This pleiotropic phenotype reflects adaptive remodeling of the cell surface: truncation of the LPS core reshapes surface charge distribution and hydration dynamics, and may promote near-surface adhesion and coordinated multicellular behaviors while constraining smooth phage engagement and genome delivery. Importantly, flagellar swimming and type IV pili-mediated twitching motility were unaffected, indicating selective modulation of matrix-associated and surface-coordinated traits rather than core motility machinery. Comparable LPS-associated trade-offs have been described in *Salmonella*, *E. coli*, and *Pseudomonas aeruginosa*, where mutations in LPS biosynthesis genes markedly alter biofilm formation kinetics and surface-associated behaviors (39, 44). Although LPS core mutations are often associated with reduced motility, the phenotype observed here is consistent with a broader model in which perturbation of LPS structure rebalances motile and sessile lifestyles rather than uniformly suppressing motility. In our system, *rfaF*-dependent LPS remodeling preserves flagellar function while promoting a hyper-swarming, biofilm-prone state, consistent with the established role of flagellar motility in the early stages of *Salmonella* biofilm development (45). While our study reveals *in vitro* phenotypic trade-offs associated with *rfaF* mutations, deep-rough LPS phenotypes caused by incomplete inner-core heptosylation are known to impose substantial fitness costs *in vivo*. Previous studies show that *rfaF* mutants in *Salmonella* and *E. coli* are more susceptible to bile salts and serum complement and display reduced virulence in animal models, supporting the importance of LPS core integrity for outer membrane stability. The hyper-biofilm phenotype observed here may represent a compensatory response to envelope stress. Thus, although *rfaF*-mediated phage resistance confers advantages *in vitro*, these mutations likely constrain bacterial fitness in host environments while potentially increasing susceptibility to host defenses (39, 44). Importantly, these results indicate that PSA5-1 resistance is achieved through distinct evolutionary routes—porin loss or LPS core remodeling—that balance immediate survival against long-term fitness consequences, reshaping surface physiology in ways that both constrain phage infection and expose new vulnerabilities exploitable for precision phage-based therapy.

By integrating extracellular infection assays with comparative genomic analyses, we speculate that susceptibility to phage PSA5-1 across *Salmonella* isolates emerges from the combined effects of surface receptor compatibility and intracellular immunity, rather than from a single dominant determinant. At the extracellular level, resistance was primarily associated with variation in the OMP OmpC and LPS architecture. Disruption or divergence of *ompC* blocked infection at the adsorption stage. In contrast, alterations in LPS composition—which serves as a secondary receptor—modulated downstream engagement, including efficient binding, tail contraction, DNA injection, and productive infection (24). These observations underscore how *ompC* and O-antigen LPS receptor microdiversity can stratify host range and adsorption kinetics even among isolates carrying ninety-seven percent conserved porin scaffolds. However, strains S6, which carry the divergent loop, exhibited reduced infection efficiency, indicating that receptor compatibility alone was insufficient to predict infection outcome. Several isolates supported efficient PSA5-1 adsorption, yet failed to sustain replication, revealing intracellular defense systems as a critical second checkpoint. Comparative genomic analysis identified a diverse repertoire of antiviral defenses, distributed unevenly across the twelve genomes. Isolates enriched in layered defense combinations strongly suppressed PSA5-1 propagation despite intact receptor access, whereas defense-poor strains supported robust lytic infection. This decoupling of adsorption from replication explains PSA5-1’s broad, yet uneven, host range. Defense systems were frequently organized within genomic hotspots flanked by mobile elements and prophage remnants, reflecting their modular evolution via horizontal gene transfer. Consistent with the previous findings, antiphage defense systems enriched within bacterial defense islands markedly suppress phage production by blocking early infection steps, degrading phage genomes, or triggering abortive infection pathways that prevent virion assembly (46, 47). Prophage carriage further modulated infection dynamics, as strains harboring higher proportions of intact prophages displayed enhanced resistance, consistent with superinfection exclusion and prophage-encoded defense functions. Previous studies demonstrate that prophage carriage profoundly modulates infection dynamics by providing superinfection exclusion, altering host surface receptors, reshaping cellular physiology, and engaging in interprophage competition that influences phage replication, induction, and population-level spread (48). Our comparative genome analysis robustly identifies defense systems and prophages using *in silico* approaches, yet the link between these defense burdens, prophages, and phage resistance remains associative. Definitive proof would require experimental validation, such as targeted knockouts or heterologous expression of defense islands, to confirm their role in restricting PSA5-1 replication. Nevertheless, the observed patterns align with known mechanisms by which RM, BREX, and related systems block phage infection (26, 47). Collectively, these findings establish PSA5-1 susceptibility as a composite trait shaped by receptor accessibility, envelope integrity, intracellular defense density, and prophage context. This multilayered resistance landscape explains why genetically similar *Salmonella* isolates exhibit divergent infection phenotypes and highlights the importance of evaluating adsorption, EOP, and phage production together when assessing therapeutic potential. From an applied perspective, these results emphasize the need for receptor-diverse phage cocktails and genome-informed phage selection strategies to overcome host heterogeneity and improve the robustness of phage-based interventions against MDR *Salmonella*.

## MATERIALS AND METHODS

### Bacterial strains and growth conditions

*S. enterica* Typhimurium S12 (ATCC 25241) was obtained from the American Type Culture Collection and cultured in Luria–Bertani (LB) broth or on LB agar at 37°C under aerobic conditions. Eleven additional *Salmonella* isolates used for host-range analysis were obtained from poultry samples collected in Dalian, Liaoning Province, China. Bacterial glycerol stocks were stored at −80°C and revived in LB broth. The appropriate antibiotics were used at the following concentrations: chloramphenicol (5 µg mL⁻¹ for *Salmonella*, 25 µg mL⁻¹ for *E. coli*) and gentamicin (15 µg mL⁻¹ for both species). All plasmids and mutant strains used in this study are detailed in Table S8.

### Phage PSA5-1 isolation, propagation, and purification

The lytic bacteriophage PSA5-1 was isolated from chicken manure in Dalian, China, using *S. enterica* S12 as the host. The isolation and purification of phage followed previously established procedures (49). Briefly, single plaques were picked and purified through three successive rounds of plaque isolation. Well-isolated plaques were harvested, treated with 1% chloroform, and clarified by centrifugation to obtain purified lysates. Phage titers were determined by the double-layer agar method and expressed as plaque-forming units per milliliter (PFU/mL). Purified stocks were stored at 4°C in phage buffer supplemented with 10 mM MgSO_4_ and 5 mM CaCl_2_.

### Transmission electron microscopy (TEM)

Phage PSA5-1 morphology was examined by transmission electron microscopy (TEM) following established protocols (15). Briefly, purified phage lysates were diluted to ~ 1 × 10⁸ PFU/mL, and 10 µL was applied to 200-mesh carbon-coated copper grids for 2 min. Excess liquid was blotted, and grids were negatively stained with 2% (w/v) phosphotungstic acid (pH 7.0) for 2 min. Phage particles were visualized using a Hitachi HT7800 TEM (Hitachi, Tokyo, Japan) operated at 80 kV, and images were digitally acquired for morphological analysis. Particle dimensions were measured using ImageJ software (https://imagej.net/ij/).

### Phage DNA extraction and genomic characterization

Phage PSA5-1 genomic DNA was extracted using the DNeasy Blood and Tissue Kit (Qiagen, CA, USA) according to the manufacturer’s instructions. Briefly, phage lysates derived from single plaques were treated with DNase I (2 U mL⁻¹) and RNase A (20 mg mL⁻¹) at 37°C for 90 min to remove host genomic DNA, followed by proteinase K digestion at 56°C for 90 min. Enzymes were inactivated with EDTA (20 mM) and heat treatment at 80°C for 5 min. DNA was purified using spin columns, eluted in Buffer AE, and its quality was assessed by 1.5% agarose gel electrophoresis. Illumina sequencing was performed at the Chinese National Human Genome Center (Shanghai, China). Raw sequencing reads were quality-checked using FastQC v0.11.8 (http://www.bioinformatics.babraham.ac.uk/projects/fastqc) and trimmed with Trimmomatic v0.39 (http://www.usadellab.org/cms/?page=trimmomatic) (50). Genome assembly was performed using SPAdes v3.5.0 (https://github.com/ablab/spades) (51), followed by gap closure with GapFiller v1.11 (https://www.baseclear.com/genomics/bioinformatics/basetools/gapfiller) (52), and error correction using PrInSeS-G v1.0.0 (https://updeplasrv1.epfl.ch/prinses/) (53). Genome annotation was carried out using RAST (https://rast.nmpdr.org/) (54), and genome visualization was generated with Proksee (https://proksee.ca/) (55). Comparative genomic analyses were performed, and major and minor capsid protein sequences were retrieved using the NCBI database, with phylogenetic trees constructed in MEGA v11 (56). Average nucleotide identity (ANI) was calculated using PyANI, and protein clustering was performed with MMseqs2 via PhageScope (https://phagescope.deepomics.org/) (57). Antimicrobial resistance and virulence genes were screened using CARD and VFDB (58, 59), respectively, and tRNA genes were identified with tRNAscan-SE (60). The complete genome sequence of phage PSA5-1 has been deposited in GenBank under accession number PX929376.

### Determination of mutation rate and comparative genomic analysis

Mutation rate determination, phage-resistant mutant selection, and comparative genomic analysis were performed, as previously described (61). Briefly, *Salmonella* strains S12 and S4 were grown to mid-log phase in LB medium and exposed to phage PSA5-1 at a multiplicity of infection (MOI) of 4. After incubation to allow adsorption and infection, cultures were plated on LB agar to select for PSA5-1–resistant mutants. Parallel control samples consisting of bacterial suspensions without phage exposure were serially diluted and plated to determine total viable counts (CFU). Mutation rates (µ) were determined using the formula µ  =  *m*/*N*, where *m* represents the mean number of resistant mutants per culture, and *N* is the total CFU. All experiments were performed in triplicate. Phage-resistant mutants of *Salmonella* strains S12 and S4 were isolated on sodium citrate agar plates. Resistance to PSA5-1 was confirmed by cross-streak assays and three successive spot tests. Verified resistant clones were cultured, preserved in 24% glycerol, and stored at −80 °C. Genomic DNA from resistant mutants was extracted using the Wizard Genomic DNA Purification Kit (Promega, USA), and DNA quality was assessed by NanoDrop spectrophotometry. Whole-genome sequencing was performed using Illumina paired-end technology at Sangon Biotech (Shanghai, China). Read quality control, trimming, assembly, gap filling, error correction, and genome annotation were conducted as described above. Assembled genomes were aligned to the corresponding wild-type S4 or S12 reference genomes using the Burrows–Wheeler Aligner (BWA) following Genome Analysis Toolkit (GATK) best practices workflow (62, 63), with duplicate reads removed using MarkDuplicates (https://gatk.broadinstitute.org/). Single nucleotide polymorphisms (SNPs) and small insertions/deletions were identified, read depth was assessed with BEDTools v2.28.0 (64), and variant effects were annotated using SnpEff. High- and moderate-impact mutations were manually curated and mapped to chromosomal loci to identify genetic changes associated with PSA5-1 resistance (65).

### Bacterial DNA manipulation and complementation

To generate chromosomal disruption of *ompC* and *rfa*F in *Salmonella* strain S4, the λ-Red recombination system was employed. Briefly, S4 cells harboring the temperature-sensitive plasmid pKD46 were grown at 30 °C, and a chloramphenicol resistance cassette (cat) flanked by 50-bp homology arms corresponding to *ompC* and *rfaF* was PCR-amplified from plasmid pKD3. The resulting PCR products were electroporated into electrocompetent S4 cells using a Gene Pulser system (Bio-Rad; 2.5 kV, 25 µF, 200 Ω). Following recovery in LB medium at 30°C for 1 h, recombinants were selected on LB agar containing chloramphenicol (10 µg mL⁻¹). Putative mutants were verified by colony PCR using primers flanking the target loci. An S4 strain carrying the empty pKD46 plasmid was included as a control. For genetic complementation, the *rfaF* gene was amplified from wild-type S4, and *ompC* was amplified from strain S12. PCR products were digested with EcoRI and XbaI and cloned into the arabinose-inducible expression vector pHB20TG. The resulting plasmids were introduced into the corresponding phage-resistant mutants, Phisa1-R3 (*rfaF*) and Phisa1-R8 (*ompC*), respectively. Transformants were selected on LB agar supplemented with gentamicin (15 µg mL⁻¹), and gene expression was induced with 0.4% (w/v) L-arabinose prior to phenotypic and phage infection assays. All mutant and complemented strains were confirmed by sequencing and preserved in 24% glycerol stocks at −80 °C. Primers and plasmids used in this study are listed in Table S8.

### Phage adsorption assay

To evaluate phage adsorption and determine the contribution of *ompC* and *rfaF* to PSA5-1 binding, time-dependent adsorption assays were performed, as previously described. Overnight cultures of *Salmonella* strains S12 and S4, along with their corresponding phage-resistant derivatives Phisa1-R8 and Phisa1-R3, were diluted 1:100 into fresh LB medium and grown to mid-log phase (OD₆₀₀ ≈ 0.35) at 37°C. Phage PSA5-1 was added at a multiplicity of infection (MOI) of 0.001 and incubated at 37 °C without shaking. For comparative adsorption efficiency, samples were collected after 20 min, centrifuged at 16,000 × *g* for 2 min at 4°C, and the number of unadsorbed phages in the supernatant was quantified by double-layer agar assay using the respective host strains. Adsorption efficiency (%) was calculated as: Adsorption (%) = (Initial phage titer − Unadsorbed phage titer)/Initial phage titer × 100. Subsequently, to assess adsorption kinetics and the direct roles of *ompC* and *rfaF*, time-resolved adsorption assays were performed using S4, S4/pKD46, S4 Δ*ompC*, S4 Δ*rfaF*, and Phisa1-R3. Samples were collected at 4, 8, 12, 16, and 20 min post-infection. To obtain cell-free supernatant, the bacterial cell suspension was centrifuged at 16,000 × *g* for 2 min at 4 °C together with adsorbed phages, a condition routinely used in phage adsorption assays and not known to dislodge reversibly bound phages. Under these conditions, both reversibly and irreversibly bound phage particles co-sediment with the bacterial pellet, ensuring accurate quantification of unadsorbed phage particles. Unadsorbed phage particles were quantified by plaque assay with the host strain. All experiments were conducted in triplicate, and results are presented as mean values ± standard deviation.

### Phenotypic adaptation associated with PSA5-1 resistance

Colony morphology of *Salmonella* strains S12 and S4, their PSA5-1-resistant mutants (Phisa1-R8 and Phisa1-R3), and S4 derivatives (Δ*ompC* and Δ*rfaF*) was assessed on LB agar following incubation at 37°C for 72 h. Colony size and morphology were documented and quantified using ImageJ. Biofilm formation for S4 was quantified using a crystal violet assay. Overnight cultures were adjusted to OD₆₀₀ = 0.4, incubated statically in LB at 37°C for 72 h, washed to remove planktonic cells, stained with 0.4% crystal violet, and solubilized with 75% ethanol. Biofilm biomass was measured at 600 nm. Motility phenotypes for S4 were evaluated to assess surface-associated behaviors. Swarming and swimming motility were assessed on 0.5% LB agar, and twitching motility on 1.5% LB agar plates, followed by crystal violet staining. Motility zones were imaged and quantified using ImageJ. All assays were performed in triplicate, and data are presented as mean ± SD.

### Phage–host interaction assays

Phage–host interactions were evaluated across twelve *Salmonella* isolates using a combination of host-range, growth inhibition, phage production, efficiency of plating (EOP), and adsorption assays. Susceptibility of *Salmonella* strain S12 and 11 additional poultry-derived isolates to bacteriophage PSA5-1 was first assessed by spot-on-the-lawn assays. Briefly, overnight cultures were mixed with molten 0.5% LB soft agar, overlaid onto 1.5% LB agar plates, and spotted with 2 µL of purified PSA5-1 to minimize variability. Plates were incubated at 37 °C for 18–24 h, and lysis patterns were recorded as clear, turbid, or absent zones. To assess infection dynamics in liquid culture, overnight bacterial cultures were diluted to an initial OD₆₀₀ of 0.01 in fresh LB medium and infected with PSA5-1 at a multiplicity of infection (MOI) of 0.1. Bacterial growth was monitored by measuring OD₆₀₀ at hourly intervals for 10 h to evaluate phage-mediated growth inhibition. Phage production was quantified by determining phage titers from culture supernatants collected at 4 h post-infection using the double-layer agar method. EOP values were calculated relative to the reference host strain S12. Phage adsorption efficiency was assessed for all 12 strains using the adsorption assay described above, and the percentage of adsorbed phage was calculated based on the reduction of free phage particles in the supernatant. All experiments were performed in triplicate, and data are presented as mean ± SD.

### Comparative genomic analysis of *Salmonella* isolates

To investigate extracellular and intracellular genomic determinants underlying differential susceptibility to phage PSA5-1, comparative whole-genome analyses were performed on 12 *Salmonella* isolates. Whole-genome sequences of strains S4 and S12 were obtained from previous sequencing efforts, while ten additional isolates (S1–S3 and S5–S11) were sequenced in this study. Genomic DNA was extracted using the Wizard Genomic DNA Purification Kit, and DNA quality was assessed by NanoDrop. Whole-genome sequencing was performed using Illumina sequencing at Sangon Biotech. Read quality control, trimming, genome assembly, gap filling, error correction, and annotation were conducted as described above. The annotated genomes were submitted to NCBI GenBank. To assess receptor-level variation, *ompC* sequences were extracted from all assemblies using BLAST-based approaches and aligned using MAFFT to evaluate sequence divergence among OmpC variants. Additionally, the physicochemical properties of the porin proteins were calculated using the ProtParam (https://web.expasy.org/protparam/). Multilocus sequence typing (MLST) was performed using the Galaxy platform, NCBI MLST, and Pathogenwatch. *In silico* serotyping, including O-antigen and flagellar antigen (H1 *fliC* and H2 *fljB*) determination, was conducted using SeqSero2 (66). Antiviral defense systems were identified using DefenseFinder (https://defensefinder.mdmlab.fr/) (67), and prophage elements were detected and classified as intact or defective using PHASTER (https://phastest.ca/).

### Statistical analysis

Statistical analyses and graph generation were performed using GraphPad Prism v9.2 (La Jolla, CA, USA). Data are presented as mean ± SD. Statistical significance was assessed using one-way ANOVA followed by Tukey’s multiple-comparison test for analyses involving more than two groups, or Student’s *t*-test for pairwise comparisons. A *P*-value <0.05 was considered statistically significant; *P < 0.05, **P < 0.01, ***P < 0.001; and ****P < 0.0001; NS, not significant.

## Data Availability

The genomic sequence of the phage PSA5-1 is available in the GenBank database under accession number PX929376. The bacterial strain S12 (ATCC 25241) was obtained from a culture collection, and sequencing data for S4 are available under BioProject PRJNA1397934. Additionally, a supplementary file containing the source data used to generate all main and supplementary figures is provided. Other datasets are available upon reasonable request.

## SUPPLEMENTAL MATERIAL

Supplemental material spectrum.00248-26-s0001.docx Tables S1, S2, and S8; Fig. S1 to S8.

Additional supplemental tables spectrum.00248-26-s0002.xlsx Tables S3 to S7.

ASM does not own the copyrights to Supplemental Material that may be linked to, or accessed through, an article. The authors have granted ASM a non-exclusive, world-wide license to publish the Supplemental Material files. Please contact the corresponding author directly for reuse.
