# Supplementary material for: Sequential receptor engagement dictates the broad host range and fitness trade-offs of Salmonella phage PSA5-1
Source: Microbiol Spectr. 2026 May 29;14(7):e00248-26. doi: 10.1128/spectrum.00248-26 (PMC13340146; doi:10.1128/spectrum.00248-26)
Supplement: Supplemental material — Tables S1, S2, and S8; Fig. S1 to S8. [file spectrum.00248-26-s0001.docx]

**Table S1. BLASTn alignments obtained using the complete nucleotide sequence of bacteriophage PSA5-1.**

| **Description** | **Query Cover** | **E value** | **Per. Ident** | **Accession** |
| --- | --- | --- | --- | --- |
| *Salmonella* phage Melville | 0.99 | 0 | 0.9962 | NC_042044.1 |
| *Salmonella* phage CF-SP2 | 0.96 | 0 | 0.9915 | OM912978.1 |
| *Salmonella* phage STML-198 | 0.93 | 0 | 0.9966 | NC_027344.1 |
| *Salmonella* phage vB_SenM-AKM_NP4 | 0.93 | 0 | 0.9838 | OR122689.1 |
| *Salmonella* phage vB_SenM_UTK0003 | 0.93 | 0 | 0.9838 | OQ359887.1 |
| *Salmonella* phage vB_SenM-S16 | 0.93 | 0 | 0.9838 | NC_020416.1 |
| *Escherichia* phage UoN_LG358_1 | 0.79 | 0 | 0.9749 | OK570374.1 |
| *Salmonella* phage vB_SnwM_CGG4-1 | 0.9 | 0 | 0.9109 | KU867307.1 |
| *Salmonella* phage vB_SnwM_CGG4-1 | 0.9 | 0 | 0.9109 | NC_031065.1 |
| *Salmonella* phage STP4-a | 0.94 | 0 | 0.9813 | KJ000058.2 |
| *Salmonella* phage STP4-a | 0.94 | 0 | 0.9813 | NC_026607.2 |
| *Salmonella* phage SEA1 | 0.91 | 0 | 0.9477 | OQ927978.1 |
| *Citrobacter* phage CkP1 | 0.5 | 0 | 0.7617 | MW239124.1 |
| *Citrobacter* phage Moon | 0.47 | 0 | 0.8023 | NC_027331.1 |
| *Citrobacter* phage CF1 ERZ-2017 | 0.45 | 0 | 0.8006 | NC_042067.1 |
| *Citrobacter* phage Merlin | 0.46 | 0 | 0.7999 | NC_028857.1 |
| *Salmonella* phage KM16 | 0.5 | 0 | 0.7988 | NC_070779.1 |
| *Enterobacter* phage CC31 | 0.28 | 0 | 0.7894 | NC_014662.1 |
| *Klebsiella* phage vB_KaeM_KaAlpha | 0.26 | 0 | 0.7885 | MN013084.1 |
| *Enterobacter* phage fGh-Ecl01 | 0.28 | 0 | 0.7882 | ON212265.1 |

**Table S2. S12 and S4 mutants resistant to PSA5-1 phage and their genotypic impact**

| **Strain** | **Chromosome POS** | **REF** | **ALT** | **TYPE** | **Samples** | **IMPACT** | **Gene function** | **Gene Bank Reference** |
| --- | --- | --- | --- | --- | --- | --- | --- | --- |
| S12 | 128 | G | A | SNP | S12_1,S12_10,S12_12,S12_16,S12_8 | MODERATE | Gifsy-2 prophage RecT | SEED:fig\|6666666.708753.peg.1907 |
| S12 | 238 | C | T | SNP | S12_10,S12_11,S12_12,S12_8 | LOW | Gifsy-2 prophage RecT | SEED:fig\|6666666.708753.peg.1907 |
| S12 | 361 | T | A | SNP | S12_1,S12_10,S12_11,S12_12,S12_13,S12_8 | LOW | Phage protein | SEED:fig\|6666666.708753.peg.1908 |
| S12 | 536944 | C | T | SNP | S12_12 | LOW | Oxaloacetate decarboxylase Na(+) pump, alpha chain (EC 4.1.1.3) | SEED:fig\|6666666.708753.peg.2630 |
| S12 | 536998 | T | C | SNP | S12_12 | LOW | Oxaloacetate decarboxylase Na(+) pump, alpha chain (EC 4.1.1.3) | SEED:fig\|6666666.708753.peg.2630 |
| S12 | 34066 | G | T | SNP | S12_16 | LOW | Flavodoxin reductases (ferredoxin-NADPH reductases) family 1 | SEED:fig\|6666666.708753.peg.35 |
| S12 | 12876 | G | A | SNP | S12_8 | HIGH | Outer membrane protein C | SEED:fig\|6666666.708753.peg.4547 |
| S12 | 331 | C | T | SNP | S12_1,S12_10,S12_11,S12_13,S12_16,S12_8 | MODERATE | Exodeoxyribonuclease VIII (EC 3.1.11.-) | SEED:fig\|6666666.708753.peg.2730 |
| S12 | 353671 | C | CG | INDEL | S12_16 | HIGH | Transcriptional regulatory protein OmpR | SEED:fig\|6666666.708753.peg.3488 |
| S12 | 353957 | TGC | T | INDEL | S12_1 | HIGH | Osmolarity sensory histidine kinase EnvZ | SEED:fig\|6666666.708753.peg.3489 |
| S12 | 353963 | TC | T | INDEL | S12_1 | HIGH | Osmolarity sensory histidine kinase EnvZ | SEED:fig\|6666666.708753.peg.3489 |
| S12 | 353965 | T | TAG | INDEL | S12_1 | HIGH | Osmolarity sensory histidine kinase EnvZ | SEED:fig\|6666666.708753.peg.3489 |
| S12 | 354661 | G | A | SNP | S12_11,S12_12 | MODERATE | Osmolarity sensory histidine kinase EnvZ | SEED:fig\|6666666.708753.peg.3489 |
| S12 | 355250 | G | A | SNP | S12_13 | HIGH | Osmolarity sensory histidine kinase EnvZ | SEED:fig\|6666666.708753.peg.3489 |
| S12 | 4431 | C | T | SNP | S12_11,S12_13,S12_16,S12_8 | LOW | hypothetical protein | SEED:fig\|6666666.708753.peg.2717 |
| S12 | 4479 | A | G | SNP | S12_10,S12_11,S12_13,S12_8 | LOW | hypothetical protein | SEED:fig\|6666666.708753.peg.2717 |
| S4 | 106555 | C | T | SNP | S4_M3,S4_M4,S4_M5 | MODERATE | ADP-heptose--lipooligosaccharide heptosyltransferase II | SEED:fig\|6666666.708743.peg.2224 |
| S4 | 128 | T | C | SNP | S4_M3,S4_M5 | MODIFIER | Phage DNA invertase | SEED:fig\|6666666.708743.peg.3845 |

**Table S8. Bacterial strains, plasmids and primers**

| **Name** | **Genotype or relevant markers** | **Remarks** |
| --- | --- | --- |
| ***E. coli*** |  |  |
| DH5α |  |  |
| ***Salmonella isolates*** |  |  |
| S4 | *Salmonella* S4, isolated from chicken feed | In this study |
| Δ*ompC* S4 | In-frame disruption of Δ*ompC* for Salmonella S4 | In this study |
| Δ*rfaF* S4 | In-frame disruption of Δ*rfaF* for Salmonella S4 | In this study |
| **Plasmids** |  |  |
| pKD3 | CmR; suicide vector with an R6K origin (pir requiring) and sacB genes from Bacillus subtilis |  |
| pKD46 | Temperature sensitive 30, Alleelic exchange vector with FLP recombinase origion, Am^R^ |  |
| pHB20TG | GmR; Arabinose-induced pBAD |  |
| pΔ*ompC* | CmR; pKD46 derivates disruption *ompC* in-frame of S4 | In this study |
| pΔ*rfaF* | CmR; pKD46 derivates disruption *rfaF* in-frame of S4 | In this study |
| p*rfaF* | GmR; pHB20TG derivates containing *rfaF* | In this study |
| p*ompC* | GmR; pHB20TG derivates containing *ompC* | In this study |
| **Primer Name** | **Primer (5' - 3 ' )** | **Remarks** |
| *ompC*_1 | GTCCCTCCTGGTACCAGCTC | S12 *ompC* verification |
| *ompC*_2 | GTCAGCGCCGTAGGTGTCGC |  |
| *ompC*_3 | GATTATTCTGTATTTTTGCGGAGAATGGACTTGCCGACTGGTTAATGAGGGTTAACCAGTAAGCAGTGGCATAAAAAAGCAATAAAGGCATATAACAGAGGGTTAATAACGGATGTAACGCACTGAGAAG | S4 *ompC* disruption |
| *ompC*_4 | GTTTATCCTCATTCGAATGGACGCAAGCGTATATCAAAACGTCGTATTTGTACGCCGGAATAAGGCATGAAAAAAGGGCCCGCAGGCCCTTTAGCAACATCTTTTGCTGACTCCAGCCTACACAATCGCT |  |
| *ompC*_5 | CCCGTTGATTTTAAAAGTTTCG | phisa1-R3 *ompC* verification |
| *ompC*_6 | CATCGCTGTTTATCCTCATTC |  |
| *ompC*_7 | tttGAAttcATGAAAGTTAAAGTACTGTCCC | *ompC*-pHB20TG (S12) |
| *ompC*_8 | tttTCTAGATTAGAACTGGTAAACCAGACC |  |
| *rfaF_1* | ATCACGCCCTTGTCATAG | phisa1-R3 *rfaF* verification |
| *rfaF_2* | CCGGCATCTTTAACCTGG |  |
| *rfaF_3* | tttTCTAGAATGAAAATTTTGGTCATTGGCC | *rfaF*-pHB20TG (S4) |
| *rfaF_4* | tttAAGCTTTTAAACGCCCTCTTCCGAC |  |
| *rfaF*_5 | GCGCGCTGGAAATCGGCGAGCGCCGCAGATTGGGCCATAGCCTGCGAGAGAGGCGCTACGATCGCGCCTGGGTGCTGCCAAATTCGTTTAAATCGGCGCTGATTCCTTTCTTTGCCAATATCCCGCACCGGGATGTAACGCACTGAGAAG | S4 *rfaF* disruption |
| *rfaF*_6 | CGCCGTGCGCATCACGCCCTTGTCATAGGCCAGCGCCACGTAGCGCTCCACCATCAGCGGCCAGGCGTCTTTATCAAGGACGCGCGCATCGTTCAGCAGGCCATAGCGCATTTCGCCACGCCAGCCGGTAAGCGATTGTGTAGGCTGGAG |  |


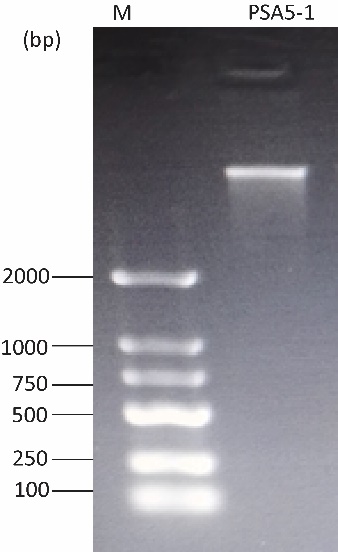


**Figure S1. Gel electrophoresis of purified PSA5-1 genomic DNA.** Agarose gel (1.5%) displaying the extracted DNA of phage PSA5-1. Lane M: DNA molecular weight marker. Lane 1: PSA5-1 genomic DNA.

**Figure S2. Sequence clustering and genomic relatedness of PSA5-1**. (A) MMseqs2 clustering of phage proteins. Protein sequences were first clustered at ≥90% identity / ≥90% coverage, and representative sequences were reclustered at ≥60% identity / ≥75% coverage to define broader protein families. The heatmap shows subcluster distribution across genomes, with PSA5-1 highlighted. (B) Whole-genome ANI heatmap generated using pyANI (ANIb). Red indicates high nucleotide identity and blue indicates low identity. PSA5-1 clusters closely with Melville-like Gelderlandvirus phages, confirming its genomic relatedness.


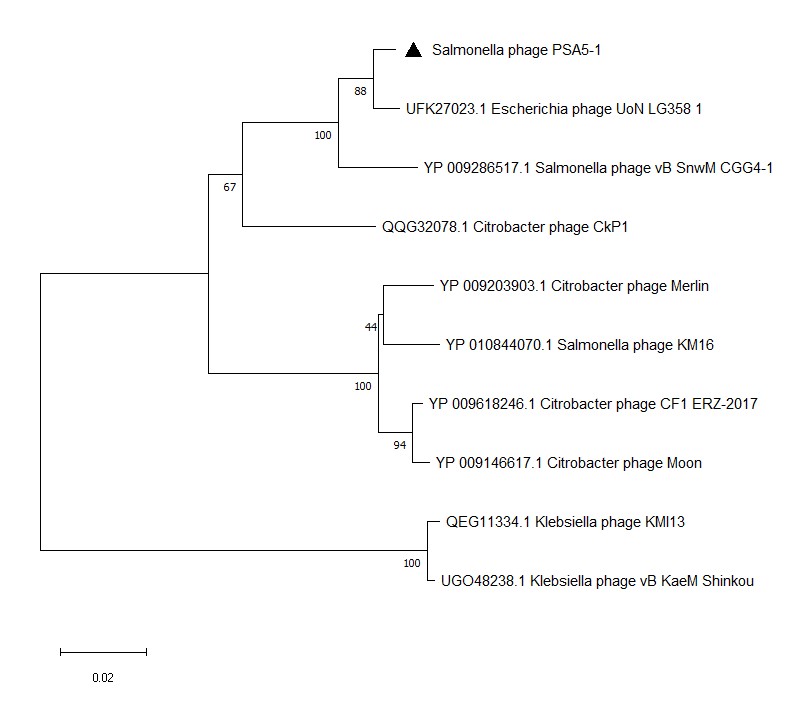


**Figure S3. Phylogenetic analysis of the terminase large subunit (TerL**). Maximum-likelihood phylogenetic tree constructed from the amino-acid sequences of the terminase large subunit. *Salmonella* phage PSA5-1 clusters in the same branch as *Escherichia coli* phage UoN LG358-1 (GenBank: UFK27023.1). This evolutionary relationship supports the classification of PSA5-1 within the family *Straboviridae* and subfamily *Tevenvirinae* (T4-like phages).


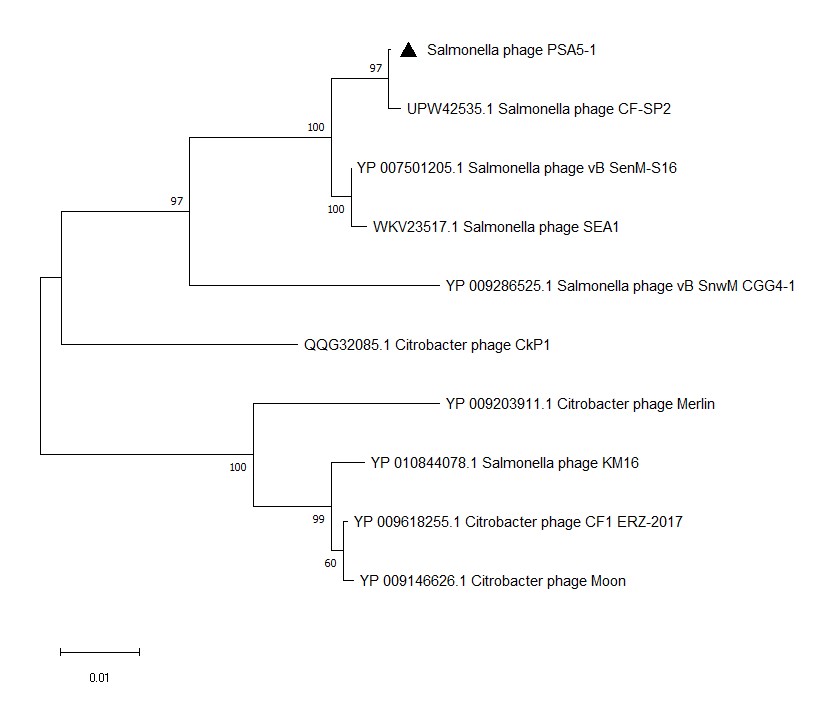


**Figure S4. Phylogenetic analysis based on the major capsid protein**. The maximum-likelihood phylogenetic tree constructed from the amino-acid sequences of the major capsid protein shows that Salmonella phage PSA5-1 clusters in the same branch as Salmonella phage CF-SP2 (GenBank: UPW42535.1). This close evolutionary relationship further supports the classification of PSA5-1 within the family *Straboviridae* (formerly part of the family *Myoviridae*) and the subfamily *Tevenvirinae*, which encompasses canonical T4-like bacteriophages.


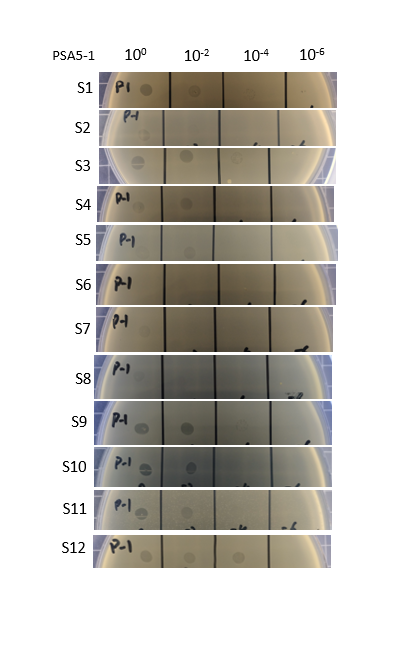


**Figure S5. Spot test of phage PSA5-1 against *Salmonella* isolates collected in this study.** Phage is almost susceptible and successfully infected 11 *Salmonella* strains except S6, demonstrating a broad host range.

**Figure S6. Spot-test confirmation of PSA5-1 resistance clones**. (A) All eight S12-derived mutants showed complete resistance to PSA5-1, with no plaque formation at any phage dilution, whereas the parental S12 strain remained fully susceptible. (B) All three S4-derived mutants similarly exhibited complete resistance, confirming the phenotype associated with the *rfaF* nonsense mutation identified by genome sequencing.


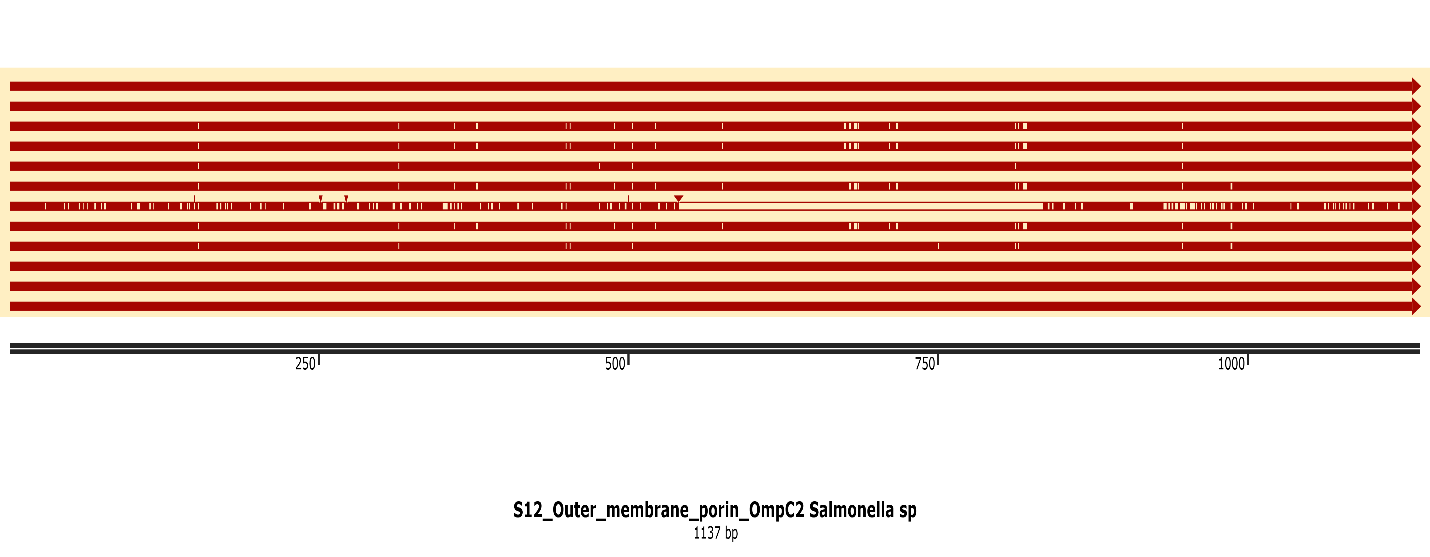


**Figure S7. Multiple sequence alignment of ompC across *Salmonella* isolates.** Alignment of *ompC* nucleotide sequences generated using SnapGene. The *ompC* sequence from strain S12 is shown at the top as the reference, followed by aligned sequences from the remaining isolates. Strain S11 lacks an identifiable *ompC* sequence, whereas strain S6 encodes a markedly divergent *ompC* variant relative to all other isolates. Conserved regions and sequence variation across the alignment are indicated.


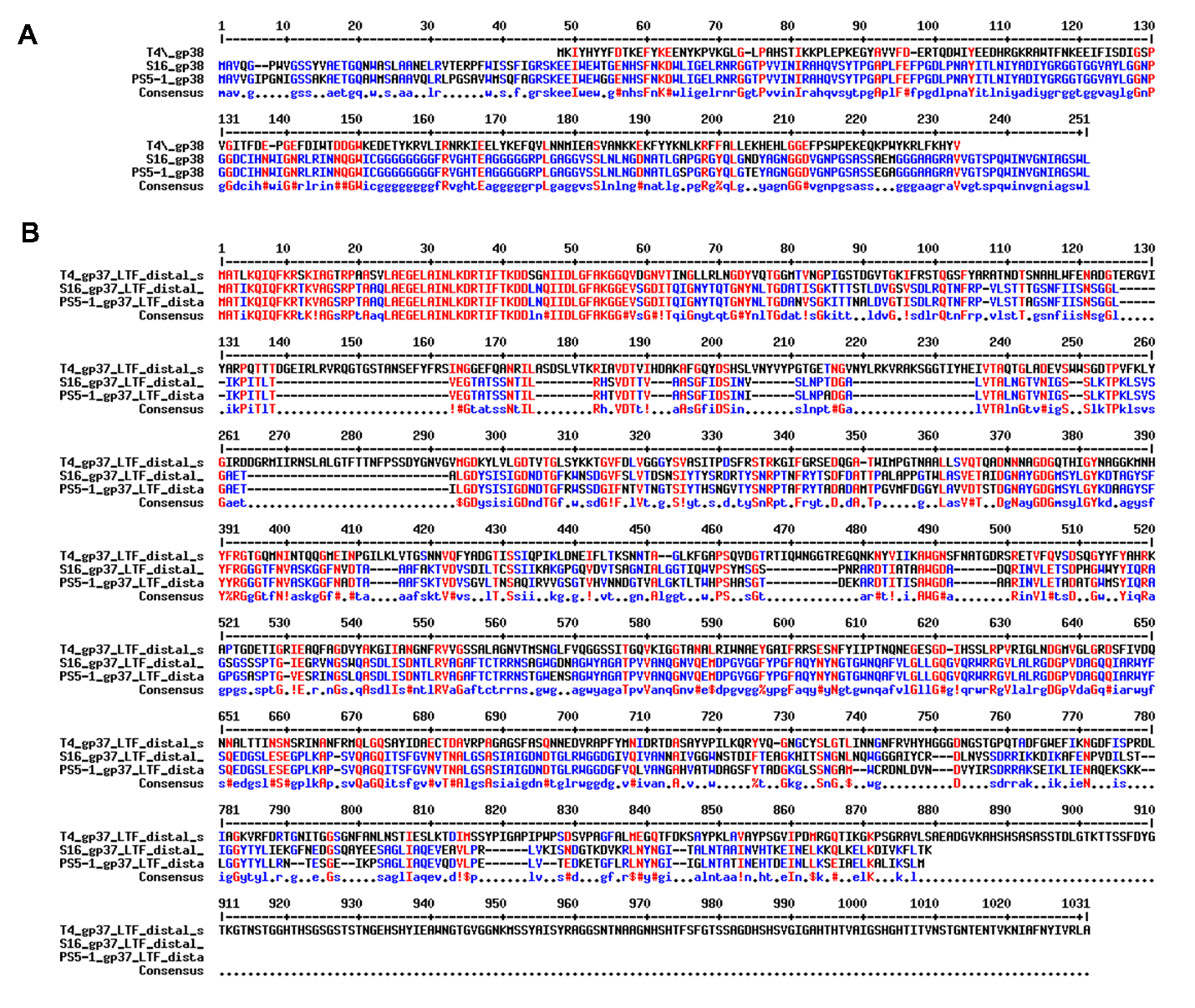


**Figure S8: Multiple sequence alignment of distal tail fiber proteins (gp38/gp37) from T4-like phages**. (A) Alignment of the gp38 receptor-binding protein from the T4, Salmonella phage S16, and phage PSA5-1. (B) Alignment of the gp37 distal tail fiber protein from the same phages. Alignments were generated using MultAlin (Florence Corpet, INRA, France), and residues are color-coded according to sequence conservation. The results show higher sequence similarity between PSA5-1 and S16 than with T4, consistent with an S16-like distal tail fiber architecture.
